# Regulation by the Transcriptional Regulator YdcI in *E. coli* and (p)ppGpp‐Dependent Activation of *ydcI* Expression

**DOI:** 10.1111/mmi.70110

**Published:** 2026-09-11

**Authors:** Ria Basu Roy, Karin Schnetz

**Affiliations:** ^1^ Department of Biology, Faculty of Mathematics and Natural Sciences, Institute for Genetics University of Cologne Cologne Germany

**Keywords:** (p)ppGpp, iraP, LTTR, LysR‐type transcription regulator, stress response

## Abstract

YdcI, a LysR‐type transcription regulator, has been associated with various phenotypes related to stress response and metabolic adaptation in *Escherichia coli and Salmonella enterica*. This work addresses regulation of specific target loci by YdcI as well as regulation of *ydcI* expression and YdcI synthesis in *E. coli* . DNA binding of YdcI to loci previously identified through ChIP‐exo analysis in *E. coli* K12 was validated for 10 loci by electrophoretic mobility shift assays. Two of these loci, *iraP* and *mqo*, are regulated by YdcI as shown using promoter *lacZ* fusions, while regulation of the other loci is marginal, at least under the conditions tested. Our data suggest that YdcI acts as an H‐NS antagonist at the H‐NS repressed and (p)ppGpp‐dependent stress‐induced *iraP* promoter. Gene *iraP* encodes the RssB anti‐adaptor protein IraP that prevents RssB‐mediated targeting of RpoS for degradation. *Gene mqo*, which is repressed by YdcI, encodes the TCA cycle enzyme malate: quinone oxidoreductase. Other YdcI bound loci encode enzymes involved in the TCA cycle, as well as membrane transport proteins. Furthermore, the transcription of the *ydcI* gene itself is induced by the alarmone (p)ppGpp under phosphate stress conditions; it is not autoregulated, and a significant increase in YdcI protein levels occurs upon entry from early to mid‐exponential growth phase. Taken together, aspects of regulation by YdcI and of *ydcI* expression as well as YdcI synthesis are presented that relate to its potential role in the response to abiotic stresses and control of metabolism in *E. coli*.

## Graphical abstract

YdcI, a LysR‐type transcription regulator in *Escherichia coli* , regulates loci like *iraP* and *mqo*. Its expression is induced by (p)ppGpp under stress conditions, and protein levels rise during mid‐exponential growth, connecting its role to stress response and metabolism control.

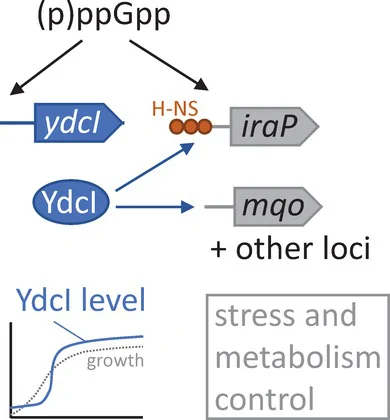

## 1 Introduction

LysR‐type transcription regulators (LTTRs), a large family of transcription regulators in bacteria, are involved in the regulation of diverse biological processes including metabolic pathways and stress responses (Henikoff et al. 1988; Baugh et al. 2023). LTTRs consist of an N‐terminal DNA‐binding domain (DBD) connected via a helical linker and a flexible hinge with a C‐terminal effector‐binding domain (EBD) that functions as regulatory domain (Baugh et al. 2023). Most, but not all LTTRs, act as tetramers that are formed by dimerization of its DBDs and of its EBDs. The LTTR's activity is assumed to be regulated by binding of a small molecule effector to the regulatory EBD or stress‐induced post‐translational modification of the EBD, but effector‐independent LTTRs have been identified (Baugh et al. 2023). A prevalent feature of LTTRs is DNA‐binding at two adjacent sites, each with a conserved T‐N_11_‐A pattern, using their two dimeric DBDs (Baugh et al. 2023). DNA‐binding per se is independent of an effector. The effector rather induces a structural change in the EBD that triggers steric repositioning of the two dimeric DBDs relative to each other resulting in their repositioning on the DNA, named sliding‐dimer mechanism, and a change of activity in gene regulation (Lerche et al. 2016; Baugh et al. 2023). Since LTTRs bind DNA specifically also in the absence of an effector their DNA‐binding sites can be identified even if no effector and/or inducing condition is known.

YdcI is conserved in *Enterobacterales* (Solomon et al. 2014) and YdcI is one of the 46 LTTRs encoded by *E. coli* K12, of which 32 (including YdcI) belong to the *E. coli* core genome (Sutton et al. 2021). Studies on YdcI have been mainly conducted in *E. coli* and *Salmonella entererica* and various phenotypes have been described (Table 1). However, conditions that modulate YdcI's protein activity and *ydcI* expression remain scarce. Briefly, YdcI in *S. enterica* autorepresses its own expression, enhances biofilm formation and confers acid stress resistance (Jennings et al. 2011; Romiyo and Wilson 2020). In *E. coli* , growth rates of an *ydcI* mutant are reduced at pH 5.5 and 8.5, but not at neutral pH, and acatete uptake is increased in the *ydcI* mutant (Gao et al. 2018). These phenotypes correlate with a presumptive role of YdcI in regulation of genes that encode proton symporters and antiporters as well as enzymes of the TCA cycle, as identified by ChIP‐exo (Gao et al. 2018). Another study revealed that *ydcI* mutants (*ydcI*‐A111E and *ΔydcI*) show an increase in the frequency of persister cells in *E. coli* (Hingley‐Wilson et al. 2020), and, in repeated long‐term starvation experiments with *E. coli* , a combination of loss of function *ydcI* mutants with a *rho‐R109H* mutation (Rho is a transcription termination factor) has been identified, and it was shown that in these double mutants the cytoplasm is alkalized (Worthan et al. 2024). In addition, a Ribo‐seq analysis suggests enhanced *ydcI* translation at pH 4.4 in *E. coli* K12 (Schumacher et al. 2023). Further, overexpression of YdcI cause reduction of cell viability and an increase of antibiotic sensitivity (Solomon et al. 2014).

**TABLE 1:** YdcI, functional roles in *S. enterica* and *E. coli* .

Feature	*Salmonella* spp.	*E. coli*	References
Transcription of y*dcI*	RpoS‐dependent, negatively autoregulated	(p)ppGpp induced, no autoregulation	Jennings et al. (2011), this work
Translation of *ydcI*	Not studied	Enhanced upon acid stress pH 4.4 (Ribo‐seq data)	Schumacher et al. (2023)
YdcI levels	Not studied	Increased upon entry to mid‐exponential growth, independent of acid stress	This work
Cell viability	No effect	Strongly reduced upon plasmidic *ydcI* expression	Solomon et al. (2014)
Antibiotic and oxidative stress sensitivity	No effect	Increased upon plasmidic *ydcI* expression	Jennings et al. (2011) Solomon et al. (2014)
Thermal stress sensitivity	No effect	Increased in Δ*ydcI* strain	Solomon et al. (2014)
Biofilm formation	Increased upon overexpression of YdcI	Not studied	Romiyo and Wilson (2020), Jennings et al. (2011)
Acid stress resistance	Decreased in Δ*ydcI* mutants	Slower growth of Δ*ydcI* mutants at low and high pH	Romiyo and Wilson (2020), Jennings et al. (2011), Gao et al. (2018)
Persister cells	Not studied	Increase in Δ*ydcI* strain	Hingley‐Wilson et al. (2020)
Alkalization of cytoplasm	Not studied	Detected in *rho‐R109H ydcI* double mutants that were isolated in long term starvation experiments	Worthan et al. (2024)

One of the targets of YdcI in *E. coli* is *iraP*, as identified by ChIP‐exo (Gao et al. 2018) and RNA‐seq (Romiyo and Wilson 2020). IraP is a small anti‐adapter protein that interacts with RssB and thus inhibits RssB‐mediated targeting of RpoS (*σ*S) for degradation by the ClpXP protease. IraP is involved in the stress response to phosphate starvation as well as during nitrogen starvation and the stationary phase (Bougdour et al. 2006). Further, *iraP* expression is activated in response to phosphate starvation conditions by (p)ppGpp, but independent of the phosphate stress specific PhoBR two‐component system (Bougdour and Gottesman 2007; Girard et al. 2018). The intracellular level of (p)ppGpp depends on the synthetase RelA, which becomes activated in response to amino acid starvation, and on the (p)ppGpp synthetase and hydrolase SpoT, which is important for the response to fatty acid, iron, phosphate and carbon starvation conditions (Gottesman 2019; Fernández‐Coll and Cashel 2020; Bange et al. 2021). Induction of *iraP* expression upon phosphate starvation is apparently mediated by inhibition of SpoT's hydrolase activity resulting in increased (p)ppGpp levels (Bougdour and Gottesman 2007). The SpoT‐dependent accumulation of (p)ppGpp in response to phosphate and fatty acid starvation as well as carbon starvation involves protein YtfK, which interacts with SpoT (Germain et al. 2019; Meyer et al. 2021).

In this work, ChIP‐exo identified DNA‐binding sites of YdcI (Gao et al. 2018) were validated by DNA‐binding analyses (EMSA), which confirmed YdcI binding to 10 loci including *iraP* as well as genes related to the TCA cycle and to membrane transport. In addition, the role of YdcI in *iraP* regulation and (p)ppGpp mediated induction of *iraP* was addressed. The data suggest that YdcI activates *iraP* presumably by acting as an antagonist of the global repressor and nucleoid‐associated protein H‐NS, which represses *iraP*. Further data suggest that *ydcI* expression itself is induced by (p)ppGpp in response to phosphate starvation (used as stress trigger) and that YdcI protein levels significantly increase upon entry to mid‐exponential growth phase, independently of acid stress. The data indicate a role of YdcI in the adaptation of the bacterial physiology for the transition from early logarithmic growth to later growth phases and to stress conditions.

## 2 Results

### 2.1 YdcI Target Loci

To characterize the functional role of YdcI we compared RNA‐seq data obtained for *E. coli* K‐12 strain U4 (Δ*lacZ*) and its isogenic Δ*ydcI* mutant U505 (Table S5) with ChIP‐exo data (Gao et al. 2018). The RNA‐seq data for YdcI, when compared with RNA‐seq data obtained in parallel for LTTRs LrhA, YafC, and YhaJ that are all expressed in the wild‐type (Figure S1), revealed statistically significant changes of transcripts levels for the majority of loci in parallel for several of the LTTRs (Table S5). We therefore focused in loci that were identified as YdcI targets by ChIP‐exo (Gao et al. 2018), for several of which significant changes in transcript levels were apparent (Table 2). These include *iraP* and *yhdV‐yhdWXYZ* that are presumably activated by YdcI, while further loci are presumably repressed by YdcI including *mqo, yobA‐yebZ‐yebY*, *lldPRD*, and *gltP*. The *iraP* gene has been identified before as an YdcI target. IraP prevents RssB‐mediated targeting of sigma factor RpoS for protein degradation (see introduction). The *yhdV‐yhdWXYZ* locus presumably encodes a lipoprotein (YhdV) and an ABC‐transporter of amino acids (YhdWXYZ) (The UniProt Consortium 2017). However, *yhdW* is a pseudogene in *E. coli* K‐12. The loci that are presumably repressed by YdcI encode the tricarboxylic acid cycle enzyme malate: quinone oxidoreductase (Mqo), the ABC transporter YobA‐YebZ‐YebY implicated in delivering copper to the copper‐dependent NADH:quinone oxidoreductase II encoded by *ndh* (Hadley et al. 2022), the GltP glutamate/aspartate:H^+^ symporter, and the *lldPRD* operon encoding the lactate/glycolate:H^+^ symporter LldP, the transcription autoregulator LldR and lactate dehydrogenase LlpD, respectively (Table 2). Additional loci identified by ChIP‐exo (Gao et al. 2018) (see Table 1) are the divergently arranged *gltA* gene and *sdhCDAB* operon, which encode citrate synthase (GltA) and succinate: quinone oxidoreductase (SdhCDAB), that is, key enzymes of the tricarboxylic acid cycle, as well as *nhaA* (Na^+^:H^+^ antiporter), *yobF* (implicated role in envelope and acid stress), *yicG* (inner membrane protein), and *dtpA* (dipeptide/tripeptide: H^+^ symporter) (Table 2). For other loci (*tomB*, *ybhI*, *wrbA*, *trkG*, and *gutM*/*srlM*) re‐evaluation of the ChIP‐exo data showed that their enrichment for YdcI binding was less strong (Figure S2). Taken together, the data analysis indicates that 11 out of the 16 predicted loci may be bound by YdcI, and for 6 of these loci significant changes in transcript levels were detected by RNA‐seq (Table 2).

**TABLE 2:** ChIP‐exo, RNA‐seq and EMSA data for YdcI.

Gene(s)	Description	ChIP‐exo^a^	EMSA^b^ YdcI_His6_	WT/ΔydcI^c^	*p*‐adj
*nhaA*	Na^+^:H^+^ antiporter	High	++	−0.19	0.614
*iraP*	anti‐RssB, *σ*S stabilization, phosphate and other stress	High	++	**2.58**	**0.000**
*gltA* *sdhC* *sdhD* *sdhA* *sdhB*	TCA cycle, citrate synthase (GltA) succinate:quinone oxidoreductase (SdhCDAB)	High	++	−0.65 0.10 −0.09 −0.21 −0.31	0.052 0.827 0.842 0.523 0.293
*yobF*	Role in envelope and acid stress	High	+	0.08	0.803
*yobA* *yebZ* *yebY*	YobA, YebZY: delivering copper to copper‐dependent NADH:quinone oxidoreductase II (NDH‐2)	High	++	**−1.04** **−0.84** **−0.63**	**0.000** **0.004** **0.007**
*mqo*	TCA cycle, malate:quinone oxidoreductase	High	++	**−1.24**	**0.000**
*yhdV* *yhdW* *yhdX* *yhdY* *yhdZ*	YhdV: lipoprotein YhdWXYZ: putative polar/charged amino acid ABC transporter (frameshift in 23rd codon of *yhdW*)	High	++	0.01 n.a. **0.90** **0.88** 0.13	0.981 n.a. **0.024** **0.032** 0.784
*lldP* *lldR* *lldD*	Lactate/glycolate:H^+^ symporter regulator L‐lactate dehydrogenase	High	++	**−0.84** **−0.90** **−1.07**	**0.010** **0.024** **0.010**
*yicG*	Inner membrane protein	High	+	−0.06	0.908
*gltP*	Glutamate/aspartate: H^+^ symporter	High	+	**−1.02**	**0.002**
*dtpA*	Dipeptide/tripeptide: H^+^ symporter	High	−	−0.02	0.955
*tomB*	Protein that modulates Hha toxicity	Low	N.D	−0.35	0.468
*ybhI*	Putative tricarboxylate transporter	Low	N.D	0.08	0.920
*wrbA*	NAD(P)H:quinone oxidoreductase	Low	−	0.48	0.343
*trkG*	Na^+^‐dependent K^+^ transporter	Low	N.D	0.23	0.501
*gutM (srlM)*	Transport and utilization of glucitol	Low	N.D	−0.23	0.669

*Note:* bold values are statistically significant.

^a^ Genes identified as YdcI bound by ChIP‐exo (Gao et al. 2018) and re‐evaluation of the data using IGV (Figure S2), with ‘high’ indicating a strong enrichment and ‘low’ indicating a minor enrichment.

^b^ Binding of YdcI determined by EMSA ++ (strong binding), + (weak binding), − (no binding), N.D. (not determined).

^c^ The log2 fold change values shown in grey are statistically non‐significant (*p*‐adj > 0.05), WT (wild‐type), not applicable (n.a.) as *yhdW* is a pseudogene in *E. coli* K12.

### 2.2 In Vitro DNA‐Binding of YdcI to Presumptive Target Loci and YdcI DNA‐Binding Motif

To determine whether YdcI binds to the presumptive target loci, in vitro electrophoretic mobility shift assays (EMSA) were performed using FAM‐labelled fragments and purified YdcI_His6_ protein (Figures 1 and 2). Specificity of DNA‐binding was assessed by competitive EMSA with unlabelled specific and non‐specific competitor fragments, respectively. Since LTTRs are generally tetramers that contact DNA at two distinct separated sites, tiling FAM‐labelled fragments overlapping by 60 bp were used to cover the respective promoter/regulatory regions.

**FIGURE 1:**
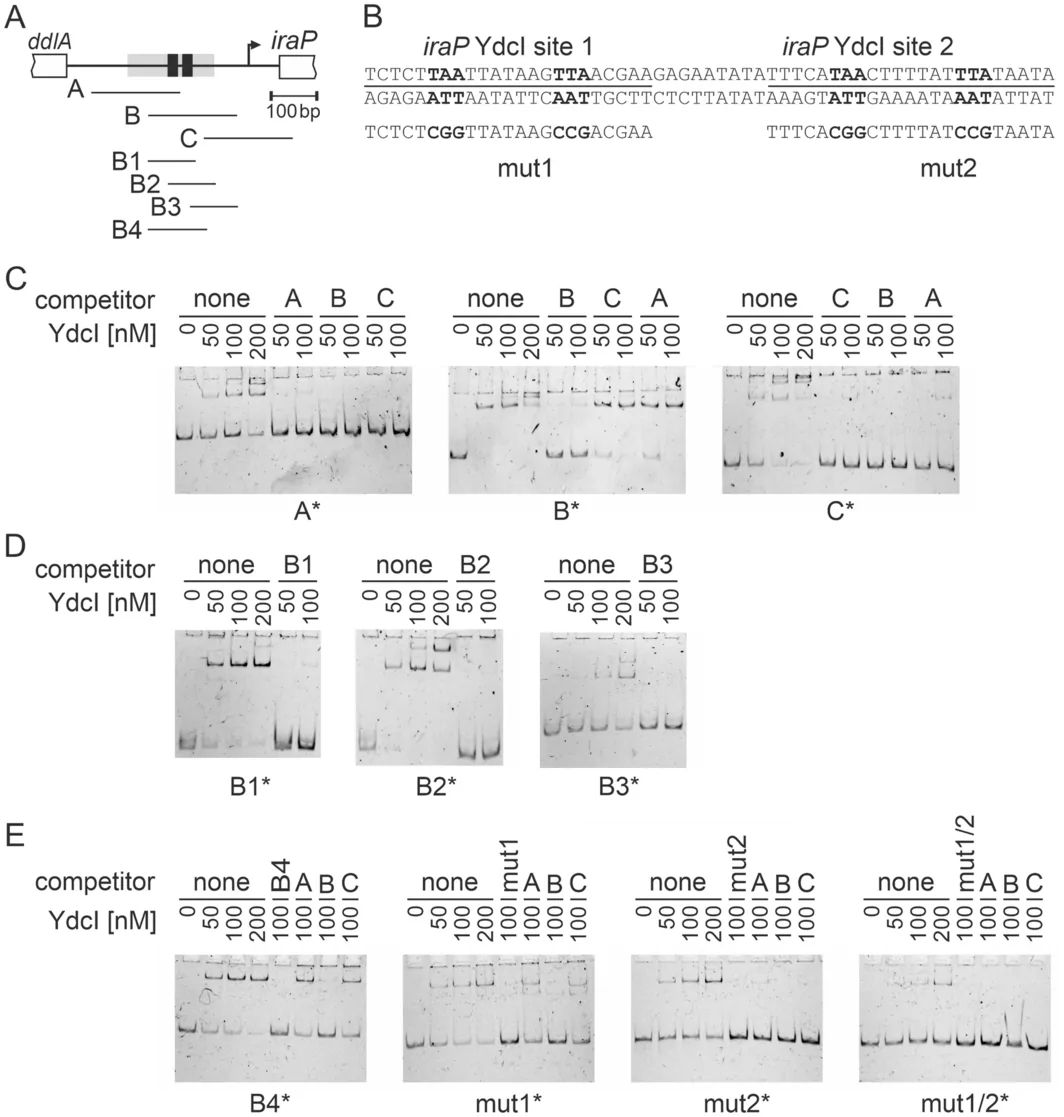
YdcI specifically binds *iraP* DNA. DNA‐binding of YdcI_His6_ was analyzed by electrophoretic mobility shift assays (EMSAs) using DNA fragments covering the *iraP* promoter‐regulatory region. (A) Schematic of the *iraP* region. DNA fragments, depicted as lines, were amplified by PCR using oligonucleotides listed in Table S4. The ChIP‐exo mapped binding site of YdcI is depicted as grey box (A) and the sequence of the YdcI motifs identified using MEME‐suite (see below, Figure 3) are indicated as black boxes (A) and shown in the sequence with binding sites ‘YdcI Site 1’ and ‘YdcI Site 2’ (B). (C to E) EMSA with increasing concentrations of purified YdcI_His6_ using (C) *iraP* fragments A, B, and C, (D) fragments B1, B2 and B3, and (E) fragment B4, as well as the fragments mut1, mut2 and mut1/2 with mutations in the YdcI sites. Unlabelled fragments A and C were used as non‐specific competitors and unlabelled fragment B was used as a specific competitor.

**FIGURE 2:**
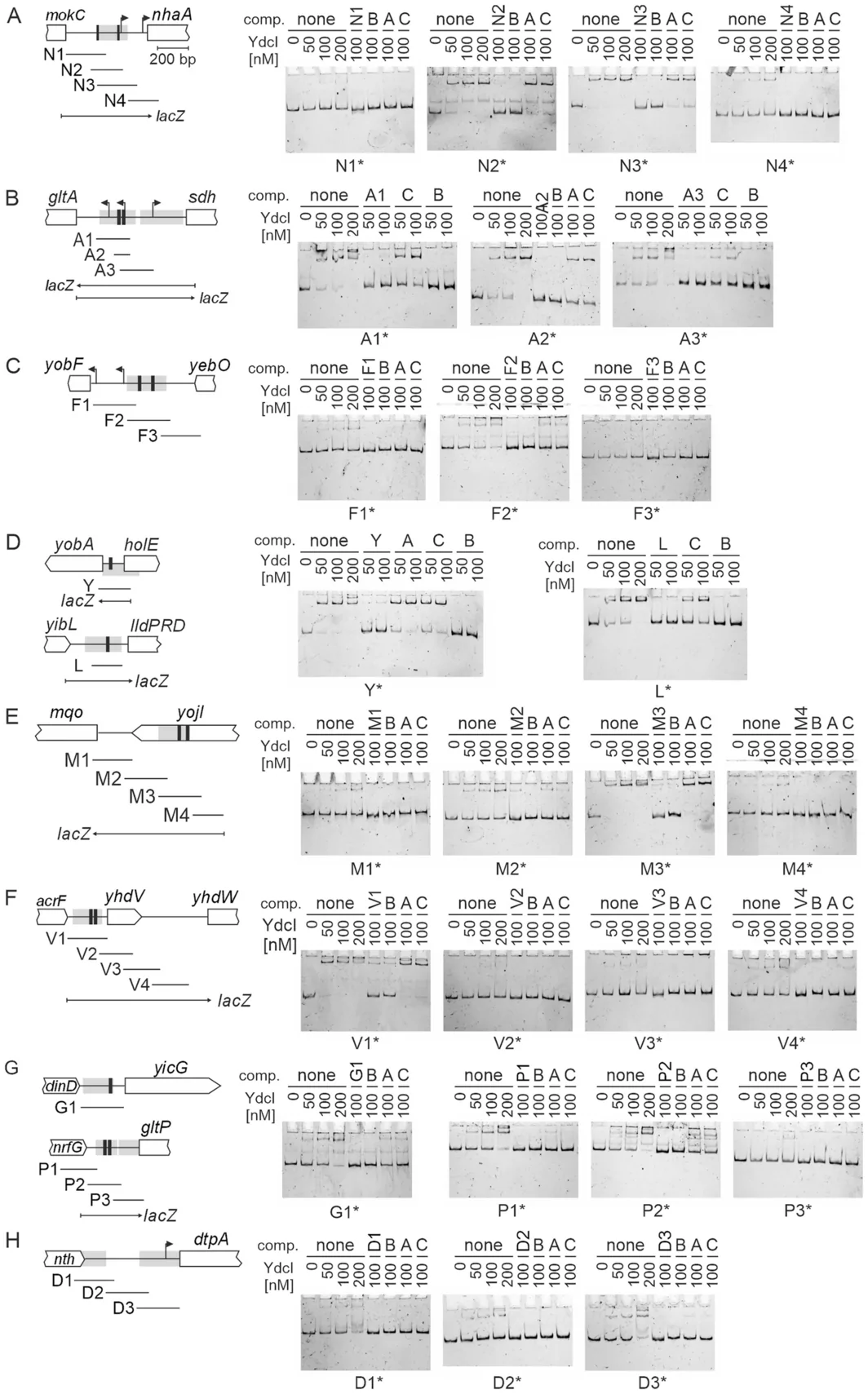
DNA‐binding by YdcI_His6_. DNA‐binding by YdcI_His6_ was analyzed by EMSA. 6‐FAM labelled and unlabelled fragments depicted in the schemes as lines were amplified by PCR using oligonucleotides listed in Table S4. The binding regions of YdcI identified by ChIP‐exo (Gao et al. 2018) are depicted as grey bars and the motifs identified by MEME‐suite (Figure 3) are indicated with black boxes. Fragments used for promoter *lacZ* fusions (Figure 4) are indictated by arrows and labelled with *lacZ*. EMSA were perfomed with increasing concentrations of purified YdcI_His6_ using FAM‐labelled fragments (marked with an asterisk *) specified below the individual gel images. Unlabbeled *iraP* fragments A and C (Figure 1) were used as non‐specific competitors and unlabelled fragment B (Figure 1) as well as an excess of the same unlabelled fragment were used as specific competitors.

In case of *iraP*, specific DNA‐binding by YdcI_His6_ was apparent for fragment B, while binding to fragments A and C was weak to non‐specific (Figure 1). Hence, for all subsequent assays, *iraP* fragment B was taken as a specific competitor, while fragments A and C were used as the non‐specific competitors. The YdcI DNA‐binding site at *iraP* was narrowed down with fragment B sub‐fragments B1 to B3. YdcI_His6_ specifically bound to subfragments B1 and B2, while binding was negligible for subfragment B3 (Figure 1D), suggesting the presence of YdcI DNA‐binding site(s) in the region overlapping B1 and B2. This map position correlates with the map position identified by ChIP‐exo (Figure 1A).

Similarly, competitive EMSA was performed using tiling fragments covering the ChIP‐exo predicted binding region for the other potential target genes of YdcI (Figure 2). These EMSA show binding of YdcI_His6_ to regions upstream of *nhaA*, *yobF*, *mqo*, *yhdV*, *lldPRD*, *yobA‐yebZY*, and to the intergenic region between *gltA* and *sdhCDAB*, while YdcI binding to *yobF, yicG* and *gltP* was observed, but with lower specificity (Figure 2). Interestingly, the YdcI DNA‐binding for *mqo* maps within gene *yojI* located upstream of *mqo*, in correlation with the ChIP‐exo data (Gao et al. 2018), indicating that the *mqo* promoter maps in the 3′ region of *yojI* (Figure 2). However, no specific DNA‐binding was detected for *dtpA* (Figure 2) although the ChIP‐exo data document two regions with a very high enrichment (Figure S2). This latter result may indicate that binding of YdcI at the *dtpA* locus requires sub‐sites mapping far apart from each other. It is also possible that in vivo additional DNA‐binding proteins support or are required for YdcI's binding to the *dtpA* regulatory region. Further, no binding of YdcI was detected for *wrbA* that was only weakly enriched by ChIP‐exo (Gao et al. 2018), and no DNA‐binding by YdcI_His6_ was detected for other loci with a statistically significant change of transcript levels in the *ydcI* mutant (log2 fold change greater than 1.5) (Table S5), indicating that these loci (including *isrC* previously reported to be regulated by YdcI based on RNA‐seq data) are not direct targets of YdcI. Taken together, the EMSA data show that only those loci significantly enriched by ChIP‐exo except *dtpA* (Figure S2) are indeed bound by YdcI_His6_ in vitro.

### 2.3 YdcI DNA‐Binding Motif

To identify a YdcI DNA‐binding motif the sequences (Table S1) of the ten YdcI_His6_ bound fragments (as shown by EMSA, Figures 1 and 2) were analyzed using MEME‐suite (Bailey et al. 2015) (Figure 3). The identified 23 bp YdcI DNA‐binding motif is AT‐rich and includes the conserved T‐N_11_‐A pattern and 15 bp core (Baugh et al. 2023). The motif is very similar to the motif identified before (Gao et al. 2018), sharing the ATAA‐N_6_‐GTTAT pattern and the AT‐rich center.

**FIGURE 3:**
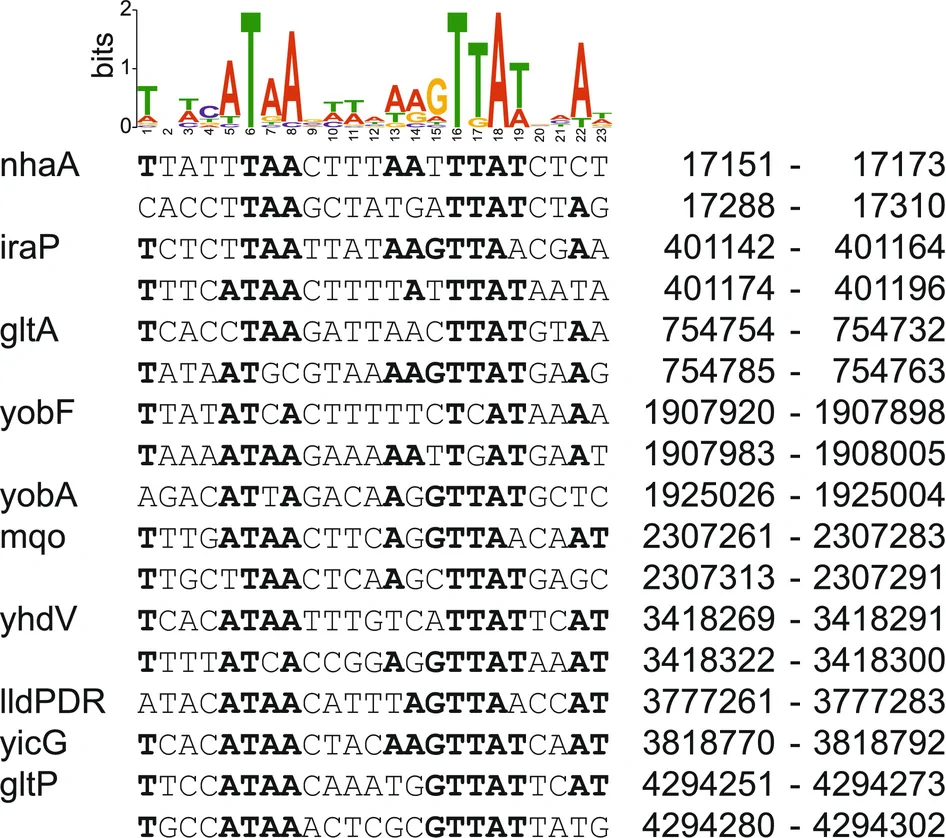
YdcI DNA‐binding motif. Top: Sequence logo for the YdcI DNA‐binding site. The logo was generated by MEME‐SUITE (Bailey et al. 2015) using the DNA sequences of the fragments that were bound by YdcI in the EMSA assays (Table S1). Bottom: Sequences of the YdcI DNA‐binding sites and their genome position in the *E. coli K12* reference genome sequence NC_000913.3. Conserved bases are depicted in upper case letters in bold.

In order to validate the YdcI DNA‐binding motif, mutations were introduced into the two YdcI DNA‐binding sites predicted for *iraP*. In fragment mut1, DNA‐binding site 1 and in fragment mut2 site 2 were mutated, while in fragment mut1/2 both DNA‐binding sites were mutated (Figure 1B). EMSA with these fragments revealed that mutation of either site reduced specific binding by YdcI_Hi6_, with mutations in site 2 showing a more pronounced effect (Figure 1E). When both sites were mutated, specific binding by YdcI was completely abolished (Figure 1E), validating the YdcI DNA‐binding sites.

At *iraP* the YdcI DNA‐binding sites map 174 to 128 bp upstream of the transcription start site and the region is AT‐rich. This location is in agreement with inhibition of H‐NS mediated repression of *P*_*iraP*_ (see below). Interesting is *lldPRD*, which is known to be autoregulated by the operon encoded LldR in dependence of lactate availability, and repressed by ArcA~*P*, the response regulator of the ArcA/ArcB two compoenent system (Lynch and Lin 1996; Aguilera et al. 2008). In addition, a posttranscriptional level of regulation controls the levels of the LldR autoregulator (Aguilera et al. 2008). At *lldPRD* the YdcI motif overlaps with the core promoter, indicative of repression by YdcI, but does not overlap with the binding sites of the other regulators. For the remaining loci the map positions of the YdcI motifs can be noted as follows (Keseler et al. 2017): At *nhaH* the YdcI‐motifs map more than 150 bp upstream of the NhaR‐activated promoter. At the *gltA*‐*sdh* locus the two YdcI motifs map in the intergenic region and one site overlaps with a presumptive ArcA binding site. At *yobF* the two YdcI motifs again map more than 127 bp upstream of the transcription start site. At *yobA* the YdcI motif maps about 50 bp upstream of the coding region and could potentially overlap with an unidentified promoter. Similar, at *mqo* YdcI may inhibit a promoter mapping within the upstream gene. At *ydhV* the YdcI motifs map 90 bp upstream of the coding region, a promoter or other transcription regulator binding sites have not been annotated. Likewise, at *yicG* the YdcI motif maps 90 bp upstream of the coding region. At *gltP* the YdcI motifs map close to the transcription start site and overlap with the core promoter and presumptive ArgR and Nac binding sites, in agreement with repression by YdcI. Interestingly, the score of the YdcI motifs at *yicG*, *yobF*, and *gltP* is as high as that of the other motifs, although fragments of these three loci are less well bound in the EMSA. This indicates that the sequence context and/or distance of adjacent motifs may affect DNA‐binding.

### 2.4 Regulation of YdcI Targets

The DNA‐binding analysis by EMSA identified 7 loci that are bound by YdcI with high specificity, while the binding specificity is lower for *gltP*, for which a significant change in transcript levels was apparent (Table 2). To analyse the regulation of these 7 loci and *gltP*, expression levels directed by transcriptional promoter *lacZ* fusions encompassing the regulatory region and first 10 codons of the gene of interest, as depicted in Figure 2, were cloned on low‐copy pSC‐derived plasmids. Expression was determined of transformants of *ydcI* ^+^ *ΔlacZ* strain U4 (referred to as wild‐type in the following) and its isogenic *ΔydcI* mutant (U505) along the growth phase over a period of 2 to 4 h. For *P*_*iraP*_ *lacZ* expression levels in the wild‐type were significantly higher than in the *ΔydcI* mutant when grown for 2 to 4 h in agreement with activation of *iraP* by YdcI (Figure 4). Furthermore, expression levels directed by the *P*_*mqo*_ *lacZ* fusions were lower in the wild‐type than in the *ΔydcI* mutant when grown for 2.5 to 3.5 h in agreement with repression by YdcI (Figure 4). For *nhaA*, *gltA*, and *lldPRD* statistically significant differences were observed for one or two time points only, which is indicative of their repression by YdcI, while no statistically significant differences were apparent for the other loci (Figure 4). These data indicate that regulation by YdcI is growth phase dependent. However, not all YdcI bound loci show regulation by YdcI. Possibly, growth in tryptone medium does not reflect conditions at which YdcI activity is induced. However, growth in tryptone media at pH 5.5, pH 7.5, and pH 8.5 yielded similar results. In the following sections regulation of *iraP* was analyzed in more detail followed by analysis of regulation of the *ydcI* promoter and determining YdcI levels along growth.

**FIGURE 4:**
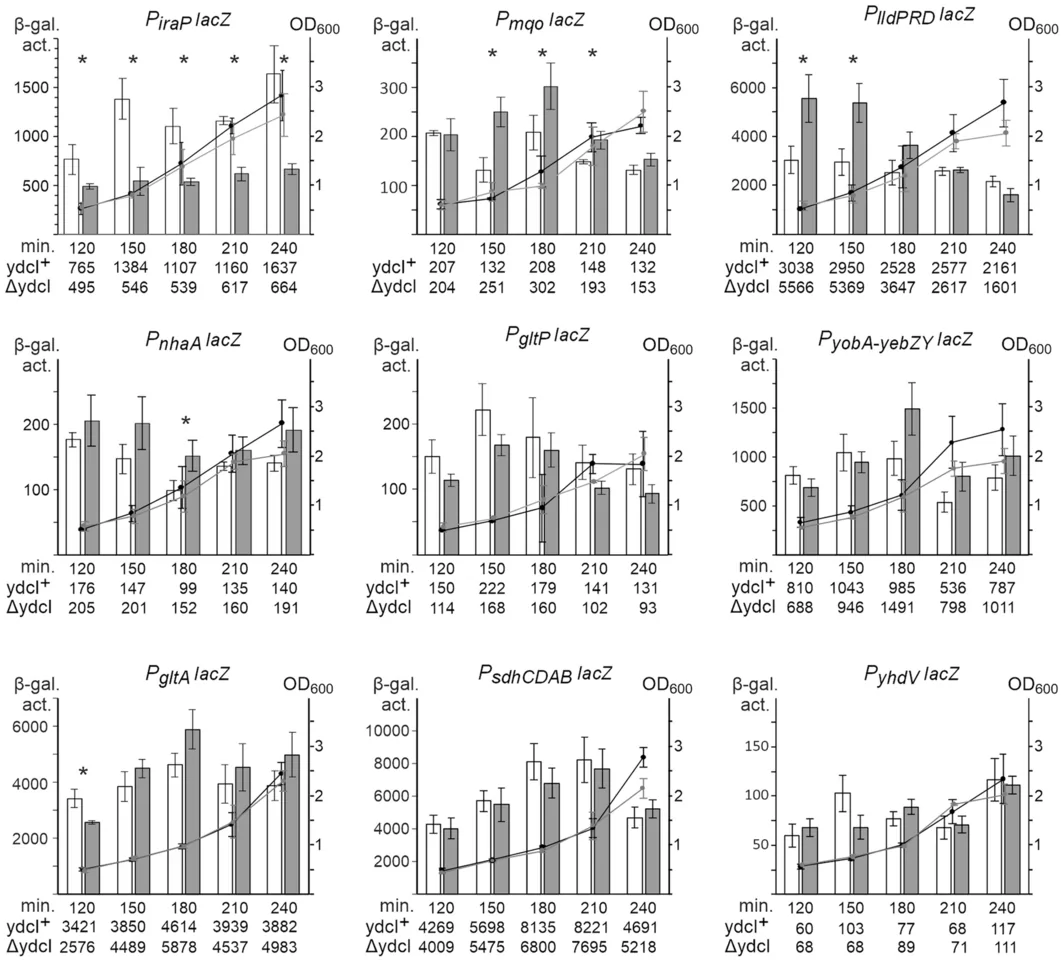
Regulation by YdcI. Regulation of promoters by YdcI was determined using plasmidic transcriptional promoter *lacZ* fusions. The fused promoter fragments are schematically indicated in Figure 2. Expression was analyzed along the growth in tryptone medium of transformants of *ydcI* ^+^ *ΔlacZ* strain U4 (black line) and its isogenic Δ*ydcI* mutant (strain U505, grey line). Plasmids used are *P*_*iraP*_ *lacZ* (pKERBR91), *P*_*mqo*_ *lacZ* (pKERBR113), *P*_*lldPRD*_ *lacZ* (pKERBR90), *P*_*nhaA*_ *lacZ* (pKERBR122), *P*_*gltP*_ *lacZ* (pKERBR123), *P*_*yobA‐yebZY*_ *lacZ* (pKERBR89), *P*_*gltA*_ *lacZ* (pKERBR111), *P*_*sdhCDAB*_ *lacZ* (pKERBR105), and *P*_*yhdV*_ *lacZ* (pKERBR112). The graphs show the growth curves as lines and the expression levels (expressed as Miller units) as bars, the time of growth is given in minutes after inoculation to OD600 = 0.08 and the average expression levels of at least three biological replicates in Miller units are stated below the graphs. Standard deviations are indicated by error bars. Statistically significant differences according to a two‐tailed *T*‐test (*p* ≤ 0.05) are marked with an asterisk (*). Differences of OD600 measurements (wild‐type versus *ΔydcI*) are not statistically significant (two‐tailed *T*‐test).

### 2.5 Regulation of the *iraP* Promoter by YdcI, H‐NS and (p)ppGpp

The *iraP* gene is one of the direct targets of YdcI, as suggested by ChIP‐exo and by RNA‐seq data, DNA‐binding assays, and expression analysis (Romiyo and Wilson 2020; Gao et al. 2018; this work). The *iraP* gene is activated by phosphate stress conditions in a DksA and (p)ppGpp dependent manner (Bougdour and Gottesman 2007; Girard et al. 2018). Further, H‐NS, an abundant nucleoid‐associated global repressor, inhibits transcription of *iraP* (Yamanaka et al. 2020) in agreement with genome wide binding profiles that identified binding of H‐NS to the *iraP* regulatory region (Uyar et al. 2009).

Here we further explored regulation of *iraP* by YdcI and H‐NS using the plasmidic *P*_*iraP*_ *lacZ* transcriptional fusion. Expression levels were determined of transformants of the *ydcI* ^+^ *ΔlacZ* strain U4 and its isogenic *ΔydcI*, *Δhns, ΔstpA* single, double and triple mutants grown to mid‐exponential phase in tryptone medium (Figure 5B). In the wild‐type expression levels directed by *P*_*iraP*_ *lacZ* are approximately 2.5‐fold higher than in the Δ*ydcI* mutant showing activation of *iraP* by YdcI (Figure 5B). Expression levels increased approximately 4‐fold in the *hns* mutant (as compared to the wild‐type) and there was no statistically significant difference in expression between the *Δhns* and *Δhns ΔydcI* mutants. The deletion of *stpA*, encoding the H‐NS paralogue StpA, did not lead to a statistically significant change in expression levels except that expression increased when comparing the *Δhns ΔstpA* double mutant and the *Δhns ΔstpA ΔydcI* triple mutant (Figure 5B). Taken together the data show activation of the *iraP* promoter by YdcI and repression by H‐NS. Further, the data suggest that YdcI counteracts H‐NS repression, since YdcI has no effect in the *hns* mutant.

**FIGURE 5:**
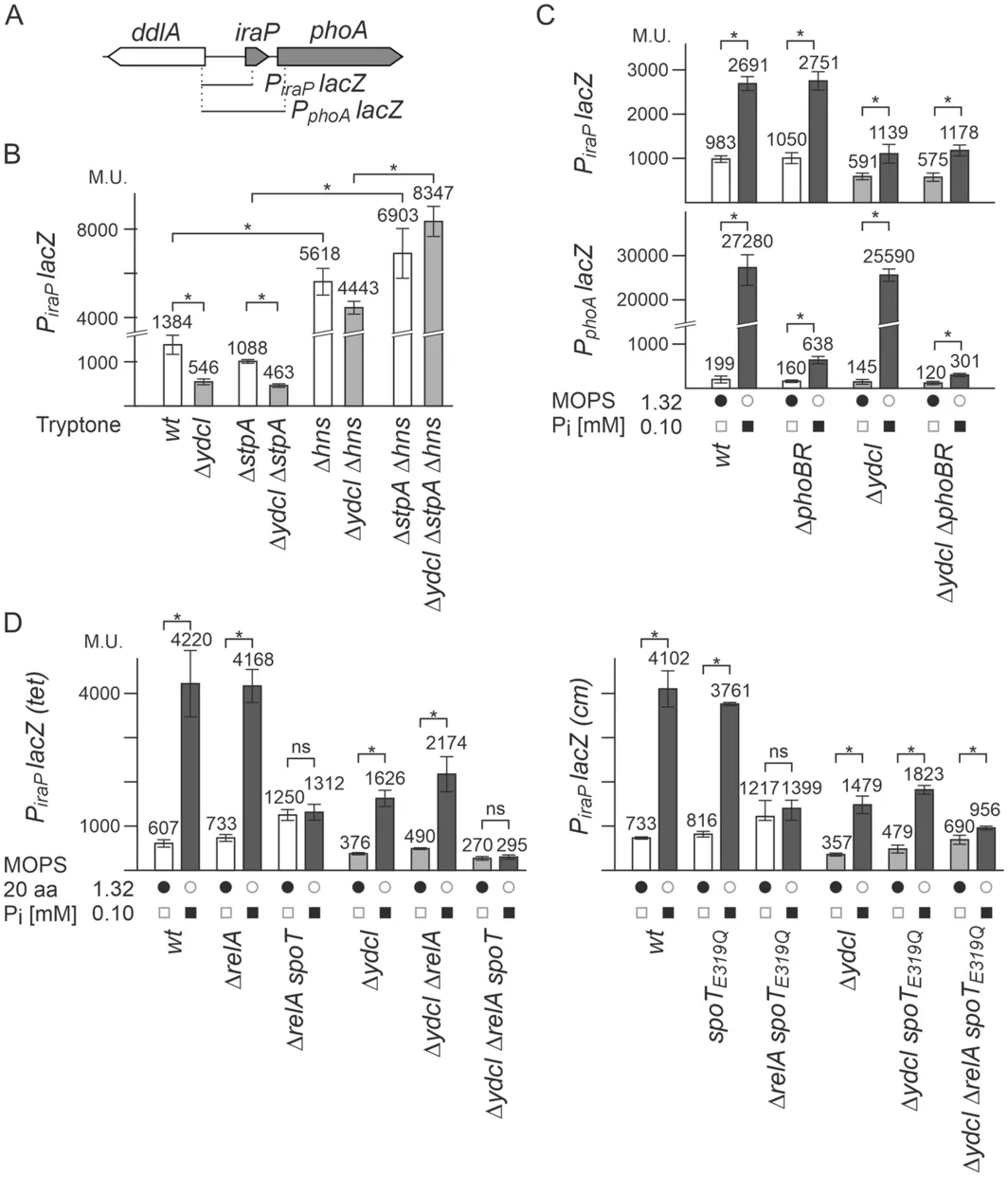
Regulation of the *iraP* promoter by YdcI, H‐NS and phosphate starvation stress. (A) Schematic of the *iraP* locus including flanking genes *ddlA* and *phoA*. The fragments used for constructing *iraP* and *phoA* promoter *lacZ* fusions are depicted by lines. (B) Expression levels directed by the *P*_*iraP*_ *lacZ* fusion (carried on low‐copy plasmid pKERBR91) in strains U4 (wild‐type, wt), Δ*ydcI* (U505), Δ*stpA* (U559), Δ*ydcI* Δ*stpA* (U560), Δ*hns* (U261), Δ*ydcI* Δ*hns* (U558), Δ*hns* Δ*stpA* (U563), and Δ*ydcI* Δ*hns* Δ*stpA* (U564) of cultures grown in tryptone medium to OD_600_ of approximately 0.8. (C) Expression levels directed by the *P*_*iraP*_ *lacZ* fusion (pKERBR91) and the *P*_*phoA*_ *lacZ* fusion (pKERBR104) of cultures grown in MOPS glucose medium with 1.32 mM K_2_HPO_4_ (black filled dots) and phosphate limiting 0.1 mM K_2_HPO_4_ (black filled squares), respectively, in strains U4 (wild‐type, wt), Δ*ydcI* (U505), Δ*phoBR* (U624), and Δ*ydcI* Δ*phoBR* (U625). Cultures were inoculated from overnight cultures in MOPS glucose 1.32 mM K_2_HPO_4_ and then grown in MOPS glucose 1.32 mM K_2_HPO_4_ and 0.1 mM K_2_HPO_4_ media to OD_600_ of approximately 0.8. Cultures carrying the plasmidic *P*_*phoA*_ *lacZ* fusion were first grown in high‐phosphate medium to OD_600_ = 0.3, then split, pelleted and washed twice with low or high phosphate medium and then grown in high and low phosphate medium to OD_600_ of approximately 0.8. (D) The regulation ofMean values from at least three independent biological replicates are shown as bars, and error bars indicate standard deviations. A Results of a two‐tailed *T*‐test are shown as ‘*’ for *p* ≤ 0.05 and as ‘ns’ for non‐significant.

Next, we tested whether YdcI affects the (p)ppGpp‐mediated phosphate stress regulation of *iraP* expression. First, *P*_*iraP*_ *lacZ* expression was increased under phosphate starvation conditions independently of PhoBR, as expected (Figure 5C). In the Δ*ydcI* mutant *P*_*iraP*_ activity was approximately 2‐fold reduced, and induction by phosphate starvation was slightly decreased from 2.5‐fold to 2‐fold in Δ*ydcI* (Figure 5C). Phosphate starvation condition comparing expression in high and low phosphate medium (MOPS glucose with 1.32 and 0.1 mM potassium phosphate, respectively) was validated using a transcriptional *phoA* promoter *lacZ* fusion (Figure 5C). The *phoA* gene, encoding alkaline phosphatase, was activated by the PhoBR two‐component system upon phosphate starvation, as expected (Gardner et al. 2019; Makino et al. 1986), while YdcI had no significant effect on *P*_*phoA*_ activity (Figure 5C). Second, induction of *iraP* was confirmed to be (p)ppGpp‐dependent and to require at least one of the (p)ppGpp synthetases RelA or SpoT, as shown before (Bougdour et al. 2006) (Figure 5D). No phosphate stress induction was observed in the *ΔrelA ΔspoT* and the Δ*relA* *spoT‐E319Q* double mutants, with *spoT‐E319Q* encoding a synthetase inactive variant (Bougdour and Gottesman 2007). Note that the cultures for analyses in the Δ*relA* and *spoT* mutants were grown in MOPS glucose medium supplemented with all 20 amino acids. Further, two variants of the low‐copy *P*_*iraP*_ *lacZ* plasmid were used, one that confers chloramphenicol resistance and one that confers tetracycline resistance, since the *spoT* alleles Δ*spoT*_*cm*_ and the synthetase defective *spoT‐E319Q* (zib563::Tn10) are tagged with tetracycline and chloramphenicol resistance genes, respectively (Figure 5D). Remarkebly, in amino acid supplemented MOPS glucose medium the fold induction by phosphate starvation of the *iraP* promoter was increased to 6‐fold in the wild‐type, while again in the Δ*ydcI* mutant phosphate stress induction of *iraP* was slightly reduced to 4‐fold and *P*_*iraP*_ activity was decreased approximately 2‐fold (Figure 5D). Taken together, the results show that the *iraP* promoter is repressed by H‐NS, activated by YdcI, induced upon phosphate starvation in a (p)ppGpp‐dependent manner and requiring RelA or SpoT, as shown before. Further, the contribution of YdcI to induction by phosphate starvation is low to moderate only.

### 2.6 The *ydcI* Promoter Is Activated in a (p)ppGpp‐Dependent Manner Upon Phosphate Starvation

Little is known about regulation of *ydcI* expression and control of YdcI activity. Here, regulation of *ydcI* transcription was studied using low‐copy plasmid pKERBR93 carrying an *ydcI* promoter (*P*_*ydcI*_) *lacZ* transcriptional fusion. First, *P*_*ydcI*_ activity was found to be neither autoregulated nor repressed by H‐NS or StpA (Figure 6A), and YdcI does not bind to its promoter DNA (Figure S3). Second, *P*_*ydcI*_ was induced by phosphate stress and this induction was independent of YdcI and PhoBR (Figure 6B), similar to phosphate stress induction of *iraP*. Therefore, the role of (p)ppGpp synthesis by RelA and SpoT in the phosphate stress induction of the *ydcI* promoter was analyzed using *relA* and spoT mutants (Figure 6C). Induction of the *P*_*ydcI*_ *lacZ* reporter was abrogated in the *relA spoT* and the *relA spoT*_*E319Q*_ double mutants, suggesting that *P*_*ydcI*_ is activated in a (p)ppGpp‐dependent manner, similar to *P*_*iraP*_ (Figure 6C).

**FIGURE 6:**
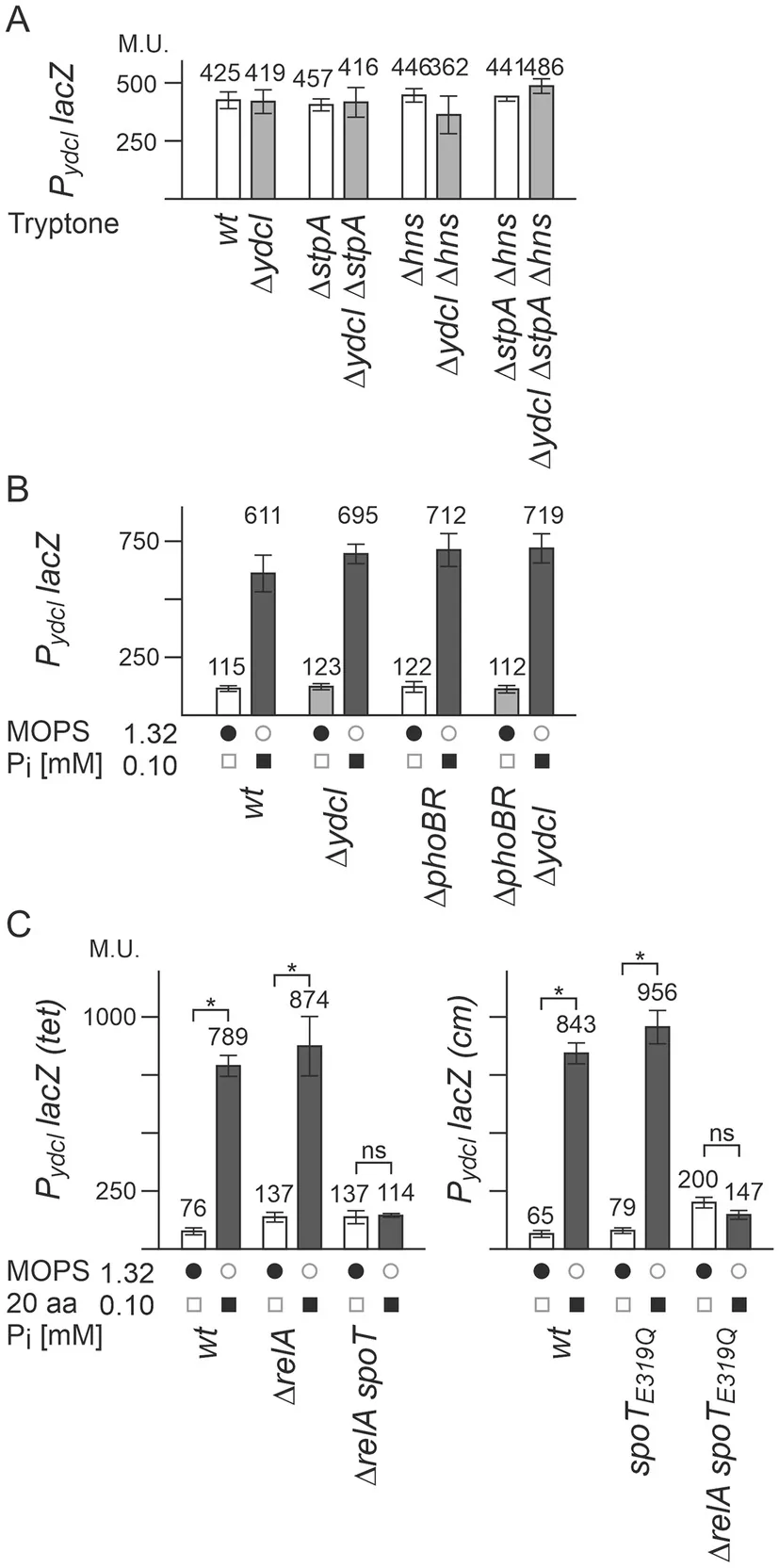
Regulation of the *ydcI* promoter by (p)ppGpp. Regulation of *P*_*ydcI*_ activity was determined using the plasmidic *lacZ* fusions *P*_*ydcI*_ *lacZ* cm (pKERBR93) and *P*_*ydcI*_ *lacZ* tet (pKERBR114). Both plasmids carry a 276 bp *ydcI* promoter region encompassing the *ydcI*‐*ydcJ* intergenic region and 10 first codons of the divergent genes. Cultures for β‐galactosidase assays were inoculated from overnight cultures and grown in (A) tryptone, (B) MOPS glucose low (0.1 mM Pi) and high (1.32 mM Pi) medium, as well as (C) MOPS glucose low (0.1 mM Pi) and high (1.32 mM Pi) medium supplemented with all 20 amino acids. Mean values from at least three independent biological replicates are shown as bars, and error bars indicate standard deviations. Statistically significant differences (*p* ≤ 0.05, two‐tailed *T‐*test) are marked with an asterisk (*). Strains wt (U4), Δ*hns* (U558), Δ*stpA* Δ*hns* (U563), Δ*ydcI* (U505), Δ*ydcI* Δ*hns* (U559), Δ*ydcI* Δ*stpA* Δ*hns* (U564), Δ*phoBR* (U624), Δ*ydcI* Δ*phoBR* (U625), as well as Δ*relA* (U571), Δ*relA* Δ*spoT* (U578), *spoT*_*E319Q*_ (U613), and Δ*relA spoT*_*E319Q*_ (U615) were used.

### 2.7 Growth Phase Dependent YdcI Synthesis

The data on regulation of promoter *lacZ* reporter fusions by YdcI indicate that YdcI activity is growth phase dependent. To analyse whether YdcI protein levels vary along growth, a strain carrying a chromosomal *ydcI‐3xFLAG* allele at the native locus was used. Western blotting showed that the amount of YdcI‐3xFLAG in LB medium increased as of the mid‐exponential growth phase and stayed high throughout growth (Figure 7A). Interestingly, a combined Ribo‐seq and RNA‐seq analysis of *E. coli* at pH stress conditions had revealed that the ribosome occupation of *ydcI* mRNA increased after a short exposure to acid stress, while its mRNA levels remained constant (Schumacher et al. 2023). To analyse the protein levels of YdcI in bacteria exposed to acid stress, YdcI‐3xFLAG levels were determined when acid stress was applied as described (Schumacher et al. 2023). Western blotting showed the level of YdcI‐3xFLAG indeed increased after exposure to 15 min pH 5.8 followed by another 15 min at pH 4.4 (Figure 7B). However, a control experiment with bacteria grown similarly at constant pH 7 demonstrated that YdcI‐3xFLAG levels similarly increase in LB medium (Figure 7B). Taken together the data show that the YdcI protein level depends on the growth phase and is independent of acid stress.

**FIGURE 7:**
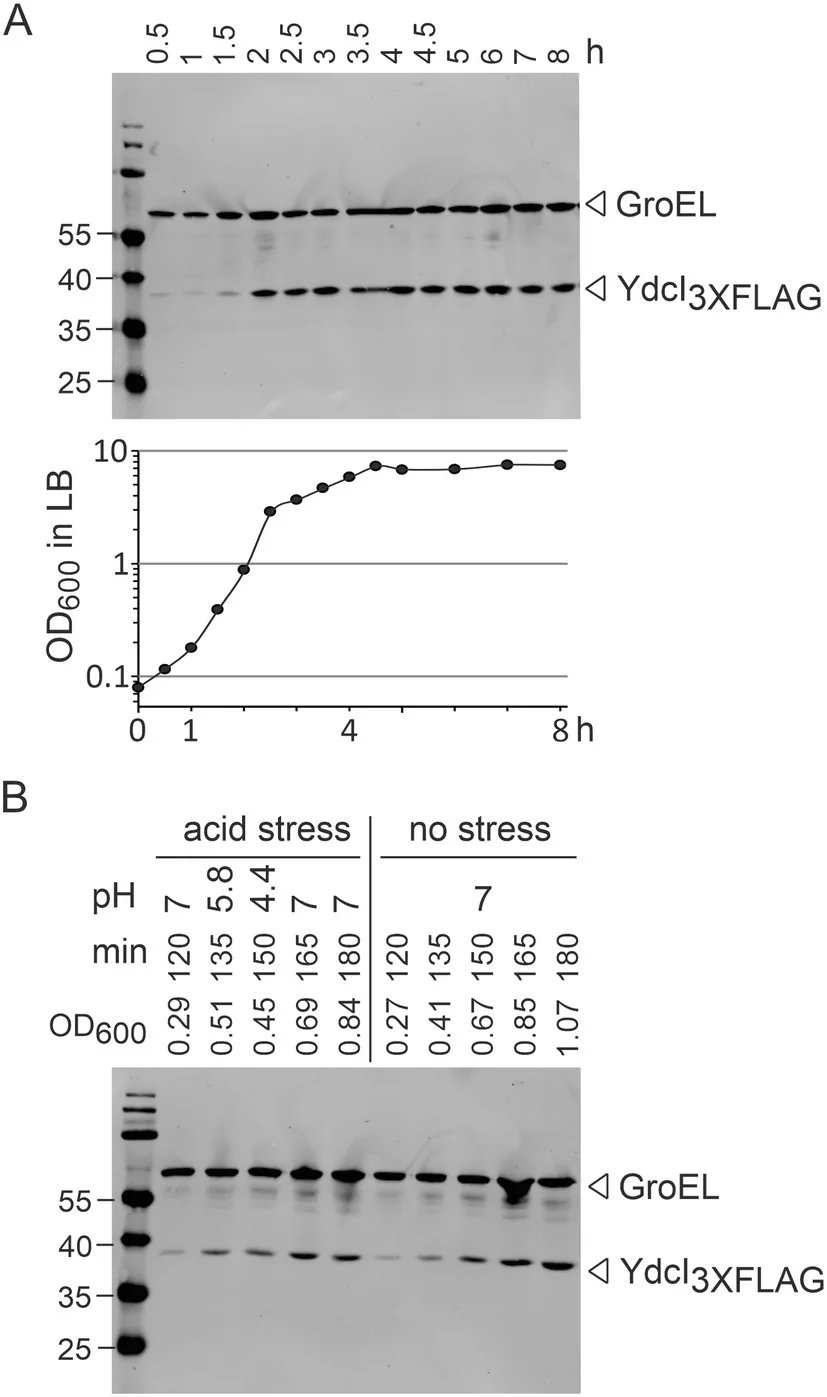
YdcI‐3xFLAG level is growth‐dependent. YdcI‐3XFLAG levels were determined along growth in LB medium and upon acid stress by Western blotting. (A) Cultures of *ydcI‐3xFLAG* strain U584 were grown in LB, and samples were harvested along growth, as indicated. The corresponding growth curve is shown underneath the gel. (B) To assess the effect of acid‐stress on the YdcI‐3XFLAG level, two cultures of ydcI‐3xFLAG strain U584 were grown in LB for 180 min. Acid stress was applied stepwise to one of the cultures by adding HCl to lower the pH from pH 7 to 5.8 for 15 min and subsequently to pH 4.4 for an additional 15 min, after which the pH of the medium was adjusted back to pH 7. As a control, the second culture was grown in parallel in LB without stress. (A, B) Bacterial pellets were resuspended in Lämmli buffer and an equivalent of 0.08 OD_600_ were loaded per lane. For the representative Western blot, primary monoclonal Anti‐FLAG M2 (1:2000, Sigma F3165) and anti‐GroEL (Abcam ab82592, 1:5000) antibodies, and secondary Goat Anti‐Mouse IgG H&L (Alexa Fluor 680) antibody (1:10,000) were used. Imaging of protein bands was performed with the Odyssey Imaging system and V3.0 software.

Since, YdcI levels increase during the early‐mid exponential phase (Figure 7A), it was investigated if the growth dependent expression of YdcI conferred any possible phenotype for YdcI. Hence, for both wild‐type and Δ*ydcI* mutant strain, a growth curve analysis was conducted in tryptone medium grown up to 4 h. The growth curves do not show any distinct difference between the *ydcI* ^+^ and Δ*ydcI* strains, but revealed a slightly longer lag phase of the Δ*ydcI* mutant of approximately 10 min as compared to approximately 6 min of the *ydcI* ^+^ upon the entry to mid‐exponential growth (Figure 8). Such a lag phase has been reported before (Wang and Koch 1978).

**FIGURE 8:**
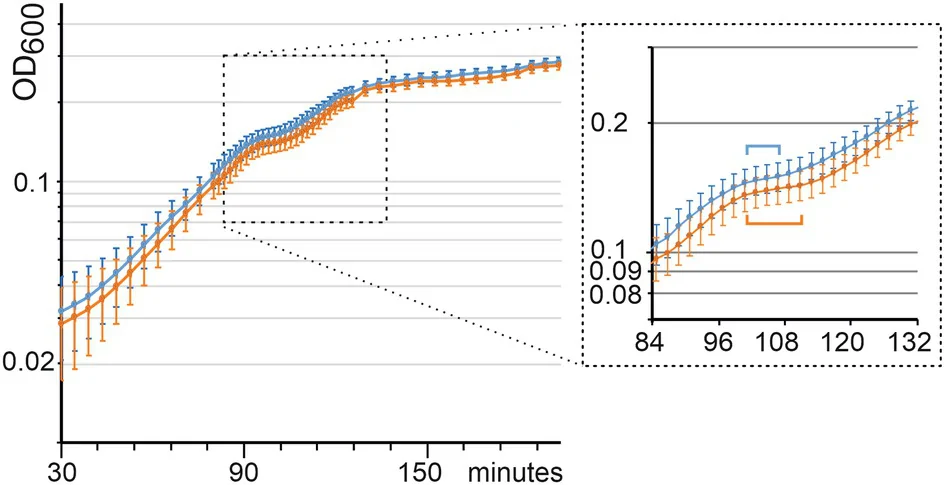
Growth analysis of *ydcI* ^+^ and Δ*ydcI* strains. Growth measurement of wild‐type strain U4 (in blue) and its isogenic Δ*ydcI* mutant U505 (in orange) in tryptone medium. The cultures were inoculated to an OD_600_ of 0.05 and the growth was monitored using a *Tecan Spark multimode reader* upto 4 h. Shown is the average of two independent experiments each of which was run in technical duplicates.

## 3 Discussion

The conserved LTTR YdcI has been associated with various functions in *Enterobacteriaceae* including a role in the acid stress response. In this study we first validated binding of YdcI to 10 out of 16 loci identified by ChIP‐exo (Gao et al. 2018) by in vitro DNA‐binding analyses. Using these 10 loci a consensus DNA‐binding motif was proposed and validated by mutagenesis of YdcI DNA‐binding sites at *iraP*. Of the 10 YdcI‐bound loci, *iraP* is activated and *mqo* is repressed by YdcI, while minor or no regulation of the remaining 8 loci by YdcI was detected using promoter *lacZ* fusions at least at the physiological conditions tested. YdcI activates *iraP* independent of its (p)ppGpp‐dependent stress induction, but presumably by antagonizing repression of *iraP* by H‐NS. Furthermore, we show that the *ydcI* promoter itself is induced by (phosphate) stress in a (p)ppGpp‐dependent manner similar to *iraP*, and that YdcI levels increase during the transition *from the early to the mid‐exponential growth phase*. Interestingly, except IraP and YobF, all other YdcI‐bound loci have a role in metabolic pathways. Taken together the data indicate that the function of YdcI relates to the stress‐triggered adaptation of metabolism (Figure 9).

**FIGURE 9:**
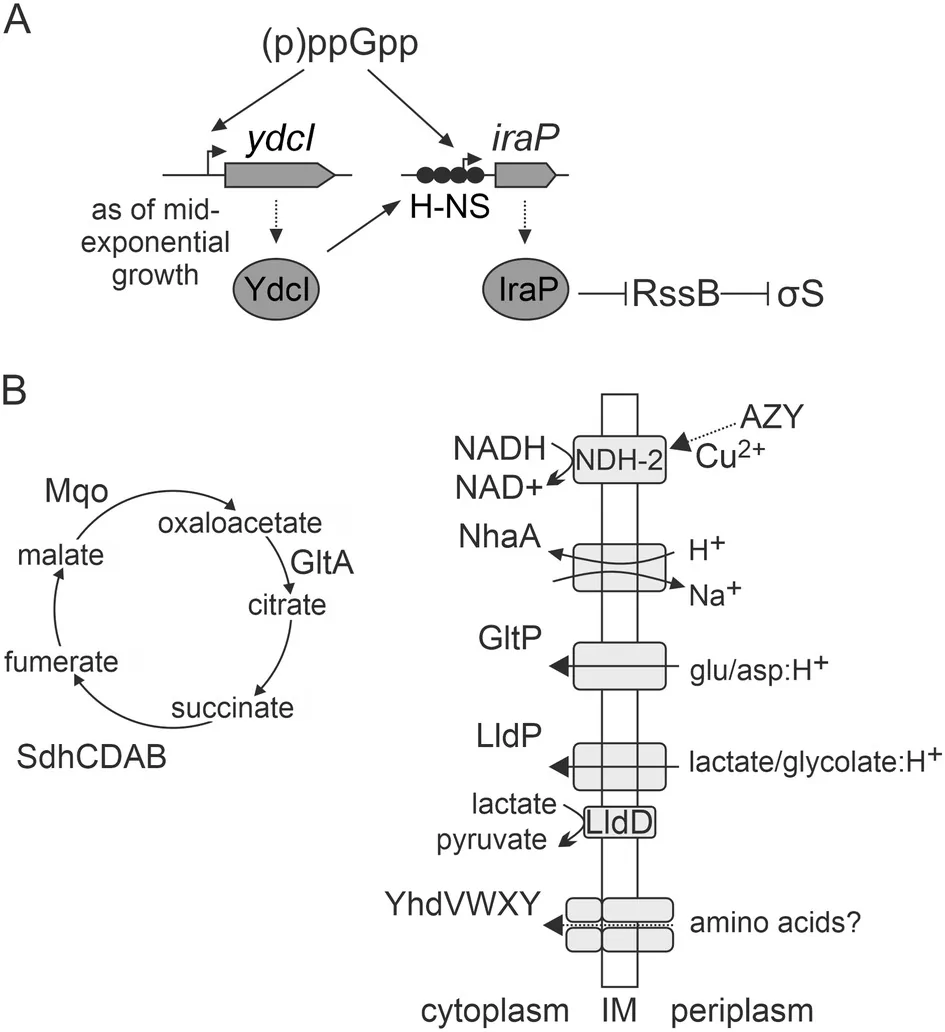
Model of transcriptional regulation of *ydcI* and *iraP* and presumptive role of YdcI in metabolic adaptation. (A) Phosphate stress and possibly other stress conditions activate the *ydcI* and *iraP* promoters in a (p)ppGpp dependent manner. H‐NS (black dots) represses the *iraP* promoter, and YdcI presumably counteracts H‐NS‐mediated repression. In addition, YdcI protein levels increase starting from the mid‐exponential growth phase. (B) The function of YdcI‐bound genes encoding enzymes of the TCA cycle, namely Mqo, GltA and SdhCDAB, and of additional loci are depicted with their encoded functions. YobA‐YebZ‐YebY (AZY) delivers copper to the NADH dehydrogenase‐II (NDH‐2) (Hadley et al. 2022). NhaA is a pH‐regulated H^+^/Na^+^ antiporter, GltP a glutamate/aspartate:H^+^ symporter, LldP is a lactate/glycolate:H^+^ symporter and LldD a L‐lactate dehydrogenase. YhdVWXY is a presumptive amino acid‐specific ABC transporter. The spectrum of YdcI targets indicates a general role for YdcI as a modulator of stress responses beyond acid stress and in metabolic adaptation.

Bacteria adapt their metabolism to the environmental condition. For example, growth in LB medium is carbon limited and bacteria grow exponentially for a short time only, followed by a gradual decline of the growth rate, presumably because bacteria then shift to catabolism of peptides, an ample component of LB medium (Sezonov et al. 2007). It has also been reported that the shift from early to the later exponential growth is accompanied by a short lag phase (Wang and Koch 1978). Such a short lag phase was also observed in this work (Figure 8) and the lag phase was slightly prolonged in the Δ*ydcI* mutant indicating that YdcI has a role in the adaptation to nutritional shifts. Furthermore, YdcI protein levels, which are very low in the early exponential growth phase, increase significantly at the same growth phase and then stay high for several hours. Upregulation of YdcI protein levels indicates regulation of translation and/or transcript stability by a small regulatory sRNA, and indeed an extensive RIL‐seq study lists interaction of the sRNA ArcZ with the middle of the coding region of the *ydcI* transcript (Melamed et al. 2020). An interesting correlation is that ArcZ levels are likewise growth phase regulated and increase at a similar time of growth as YdcI (Mandin and Gottesman 2010). ArcZ is repressed by the ArcA/ArcB two‐component system, which responds to oxygen consumption and is activated by a decrease in the electron transport chain (Brown et al. 2022). Furthermore, YdcI binding sites map to distinct locations than the DNA‐binding sites of the ArcA~*P* regulated and LLdR autoregulated *lldPRD* operon, while at the divergently arranged ArcA~*P* regulated *gltA–sdhCDAB* locus one of the YdcI motifs overlaps with a presumptive ArcA~P binding site (Lynch and Lin 1996). Additionally, the promoter *lacZ* reporter expression analysis showed a tendency of regulation of the targets of YdcI in a time‐dependent manner (Figure 4), while the slight regulation of target loci by YdcI other than *iraP* and *mqo* indicates that a small molecule effector of YdcI and/or appropriate environmental conditions for regulation by YdcI remain to be identified.

In line with the growth phase dependence of YdcI levels, all the identified YdcI target loci, except *iraP* and *yobF*, have a range of roles in different metabolic pathways (Figure 9). The YdcI‐repressed *mqo* encodes the TCA cycle enzyme malate: quinone oxidoreductase. Gene *gltA* encodes the TCA cycle enzyme citrate synthase and this enzyme's activity is increased in an Δ*ydcI* mutant (Nishio et al. 2013). Gene *gltA* maps divergently to *sdhCDAB* encoding the TCA cycle enzyme succinate: quinone oxidoreductase. In addition, the *yobA‐yebZ‐yebY* operon is required for copper binding and delivery to NADH dehydrogenase‐II (NDH‐2) (Hadley et al. 2022). The repression of *mqo* and YdcI's binding to the *gltA‐sdhCDAB* intergenic region and to the *yobA‐yebZ‐yebY* promoter indicates a potential role of YdcI in down‐regulating the carbon flux through the TCA cycle, which is possibly related to a nutritional shift from early to mid‐exponential growth. Gene *yobF*, another target gene of YdcI, is induced upon heat shock and important for acid stress response in *E. coli* (Hobbs et al. 2010), which emphasizes the potential role of YdcI in different stress conditions. Additional identified targets of YdcI include a presumptive amino acid ABC transporter (*yhdWXYZ* and lipoprotein YhdV), symporters (LldP, GltP), and the pH‐regulated NhaA H^+^/Na^+^ antiporter (implicated in maintaining cellular pH homeostasis during alkali challenge), indicating a possible role of YdcI control of transport of metabolites and in pH homeostasis (Figure 9).

Another aspect of this work is that the comparative RNA‐seq analysis of four LTTRs (namely YdcI, YafC, YhaJ, and LrhA) uncovered a wide‐ranging overlap of changes in transcript levels. LTTRs share a preference for AT‐rich DNA and a degenerate consensus DNA‐binding motif, but whether a lack of any of these LTTRs may affect DNA‐binding by other LTTRs and/or the chromosomal DNA‐binding pattern of nucleoid‐associated proteins, such as H‐NS, remains unclear. Considering these and the data of this work, identification of LTTR DNA‐binding sites by ChIP‐exo appears straightforward.

Taken together, our results validate YdcI as activator of *iraP* (Figure 9). YdcI presumably acts as H‐NS antagonist at *iraP*, since the activity of the *iraP* promoter is similarly high in the *hns* single and the *hns ydcI* double mutant. Activation by antagonisms of H‐NS, an abundant global repressor of stress and pathogenicity related genes, is a known principle of regulation (Navarre et al. 2007). Antagonism of H‐NS mediated repression of the stress‐induced *iraP* gene by YdcI might have a role in eliciting the RpoS mediated general stress response, as IraP associates with RssB and prevents ClpXP mediated degradation of the stress sigma factor *σ* ^S^ (Bougdour et al. 2006). Furthermore, activation of the *ydcI* promoter by (p)ppGpp, requiring either one of the (p)ppGpp synthetases RelA or SpoT, as shown before for *iraP* (Bougdour et al. 2008), demonstrates a link between the second messenger and alarmone (p)ppGpp and expression of the *ydcI* gene. Taking together, YdcI seems to play a role in the response to different physiological stresses beyond acid stress in *E. coli* , and potentially contribute to the adjustment of the metabolism to such environmental conditions.

## 4 Experimental Procedures

### 4.1 Bacterial Strains and Growth Conditions

*E. coli* K‐12 strains are listed in Table S2. Strains were constructed by transduction using phage P1*vir* (Miller 1992), and by Lambda‐Red‐mediated recombineering, as described (Datsenko and Wanner 2000). Bacteria were cultured in LB medium (10 g/L tryptone, 5 g/L yeast extract, and 5 g/L NaCl), tryptone medium (10 g/L tryptone, 5 g/L NaCl), SOB medium (20 g/L tryptone, 5 g/L yeast extract, 0.5 g/L NaCl, 2.5 mM KCl, 10 mM MgCl_2_, pH 7.0), SOC medium (SOB with 0.4% glucose). MOPS 0.5% glucose minimal medium is composed of 0.1 mM MOPS pH 7.4, 0.1 mM Tricine pH 7.4, 1 mM FeSO_4_, 0.19 M NH_4_Cl, 27.6 mM K_2_SO_4_, 0.5 mM CaCl_2_, 52.8 mM MgCl_2_, 0.5 M NaCl, micronutrients (30 μM (NH_4_)_6_Mo_7_O_24_•4H2O, 4 μM H_3_BO_3_, 300 μM CoCl_2_, 100 μM CuSO_4_, 800 μM MnCl_2_, 100 μM ZnSO_4_), 5 g/L glucose, and supplemented with 0.1 mM or 1.32 mM of K_2_HPO_4_, as described (Neidhardt et al. 1974; Rice et al. 2009; Bougdour et al. 2006). Antibiotics were added to a final concentration of 50 μg/mL ampicillin, 10 μg/mL chloramphenicol, 12 μg/mL tetracycline, and 15 μg/mL kanamycin, as required.

For phosphate starvation experiments, all cultures were grown overnight in MOPS glucose 1.32 mM Pi (high phosphate) medium and inoculated at an OD_600_ of 0.08 in low (0.1 mM Pi) or high (1.32 mM Pi) phosphate MOPS glucose medium. Where indicated, phosphate starvation stress was applied after cultures were grown to an OD_600_ of approximately 0.3 to 0.4 in MOPS glucose 1.32 mM Pi medium, then harvested, washed twice in MOPS glucose 0.1 mM Pi medium, and then grown in MOPS glucose 0.1 mM Pi medium. Cultures were harvested at an OD_600_ of approximately 0.5 to 0.8 for β‐galactosidase assays.

### 4.2 Plasmid Construction

Plasmids were constructed according to standard DNA cloning techniques using oligonucleotides and gene fragments listed in Table S4. As a principle, all segments of plasmids that were generated by PCR and all cloning fusion sites were sequenced. In addition, the integrity of the sizes and structure of each plasmid was validated by restriction enzyme analyses.

### 4.3 β‐Galactosidase Expression Analysis

For expression analyses of promoter *lacZ* fusions, bacteria were grown as described in the figure legends. The β‐galactosidase assays were performed as described (Miller 1992) of at least 3 independent biological replicates, and standard deviations were calculated. Statistical significance was analyzed using a student's two‐tailed *t*‐Test.

### 4.4 Growth Analysis

To measure the growth of relevant strains over time, the *Tecan Spark multimode reader* was used. Cultures were assayed in 96‐well clear bottom microplates. The strains of interest were first grown overnight at 37°C in 4 mL tryptone. The overnight culture was harvested by centrifugation and the pellet was resuspended in equal volume of Mg saline (0.85% NaCl, 10 mM MgSO_4_) followed by OD_600_ measurement for each strain. The required volume of each culture was taken to achieve an OD_600_ of approximately 0.05 in 250 μL tryptone in each well. The *Tecan Spark* measurements were taken every 5 min for 1 h 25 min, every 2 min for the next 50 min and then for every 5 min for the next 1 h 35 min.

### 4.5 Western Blotting

For determining levels of YdcI_3XFLAG_, strain U584 (U4 *ydcI‐3xFLAG*) coding for epitope‐tagged YdcI_3XFLAG_ under the control of its native promoter was used (Table S2). Cultures grown overnight were used to inoculate 20 mL of LB medium to an OD_600_ of 0.08 and grown up to 24 h at 37°C. Samples were pelleted at the time points indicated by centrifugation for 1 min at 12,000 rpm.

SDS‐PAGE and Western blot procedures were performed as described previously (Sambrook and Russell 2001). Per lane, cells at an OD_600_ of 0.08 were loaded, and equal loading was verified by 2,2,2‐trichloroethane staining of total protein (Ladner et al. 2004). Epitope‐tagged YdcI_3F_ was detected using primary antibody anti‐FLAG M2 from mouse (diluted 1:2000) (catalogue number F3165; Sigma) and Alexa Fluor 680 fluorescent dye‐labelled secondary anti‐mouse antibody from goat (diluted 1:10,000) (catalogue number A21057; Thermo Scientific) and anti‐GroEL antibody (diluted 1:5000, Abcam 82592). Quantification of protein bands was performed with an Odyssey Imaging system and V3.0 software.

### 4.6 Electrophoretic Mobility Shift Assays

DNA‐binding analyses were performed by electrophoretic mobility shift assays (EMSA). DNA fragments were amplified by PCR using oligonucleotides specified in Table S4. After clean‐up of the fragments (NucleoSpin Gel and PCR Clean‐up Mini kit, Macherey‐Nagel, Germany), the concentration of the DNA samples was estimated by agarose gel electrophoresis and the samples stored at −20°C. DNA‐binding reactions with purified YdcI_His6_ were set up on ice in 10 μL binding buffer (20 mM Tris–HCl [pH 7.5], 100 mM KCl, 2 mM DTT, 10% glycerol, 1.25 ng/μL poly[d(I‐C)]) with 2.5 nM of 5′ 6‐FAM (Fluorescein) labelled DNA fragments, and YdcI_His6_ protein at the concentration specified in the figures. Unlabelled PCR fragments were added as competitors at a concentration of 50 nM or 100 nM, as indicated. After set‐up of the EMSA samples on ice, they were incubated at 30°C for 20 min and then rapidly loaded onto an 8% polyacrylamide (acrylamide: bis‐acrylamide 29:1), 0.5× TBE (45 mM Tris‐base, 45 mM boric acid, 1 mM EDTA) gel. The gel was run in the cold room for 10 min at 50 V, followed by 55 min at 200 V. Imaging of gels with the FAM‐labelled fragments (Absorbance max: 495 nm, Emission max: 520 nm) was performed using a BioRad Chemidoc MP system (settings blue EPI light illumination, filter 530/28). Gel pictures are shown with inverted colors.

### 4.7 YdcI_His6_ Protein Purification

For purification of C‐terminally histidine‐tagged YdcI_His6_, strain C41(DE3) was transformed with plasmid pKESL317 carrying the *ydcI*_*His6*_ under control of the *T7* promoter (Table S3). For protein purification, overnight cultures of the transformant were used to inoculate 400 mL of LB medium containing 50 μg/mL ampicillin to an OD_600_ of 0.1, and the cultures were grown at 37°C for 1.5 h to an OD_600_ of 0.6. Protein expression was induced by adding IPTG to a final concentration of 200 μM, and the cultures were grown further for 1 h at 37°C. The cultures were harvested by centrifugation, cells were resuspended in resuspension buffer (20 mM Tris–HCl pH 8.0, 300 mM KCl), and pelleted for storage at −80°C. Lysate preparation was performed at 4°C; the cell pellets were resuspended in lysis buffer (4 mL/g of bacterial cells) (20 mM Tris–HCl pH 8.0, 300 mM KCl, 20 mM imidazole, 20 mg/mL DNase I [5000 U/mL] [New England BioLabs, USA]) and lysed by sonication (40% amplitude for 4 min with a 2 s pulse/2 s pause) (Sonics Vibracell VCX750 High‐Volume Ultrasonic Cell Disrupter). The lysate was cleared by centrifugation (two times; 40,000 rpm, 30 min each; Sorvall SS34 rotor). Then the cleared lysate was added on ice to 0.5 mL Ni‐NTA slurry (Protino, Machery Nagel; pre‐washed with 2 mL lysis buffer) and protein binding was allowed for 60 min on a rocking station at 4°C. Subsequently, the Ni‐NTA slurry was transferred to a polypropylene column with closed outlet. After the resin has settled, the column was washed with wash buffer (20 mM Tris–HCl pH 8.0, 300 mM KCl) with increasing imidazole concentrations (20 and 60 mM) with 10 times the resin volume at each step. The proteins were eluted with elution buffer (20 mM Tris–HCl pH 8.0, 300 mM KCl, 250 mM imidazole), and 500 μL fractions were collected and analysed by SDS‐PAGE. The eluted fractions of purified protein were stored in 20 mM Tris–HCl pH 8.0, 300 mM KCl containing 50 mM NDSB‐256. Protein concentrations were measured by Pierce bicinchoninic acid (BCA) protein assay (Thermo Scientific, Germany). Aliquots of the proteins were stored at −80°C.

### 4.8 RNA‐Seq and Data Analysis

For RNA‐seq, total RNA was isolated from strains (U4, Δ*yafC* (U456), Δ*yhaJ* (U458), Δ*ydcI* (U505) and Δ*lrhA* (U504)) with U4 taken as control. These strains were inoculated to an OD_600_ of 0.05 in 10 mL tryptone medium and grown for 2.5 h to an OD_600_ of approximately 0.8 at 37°C with shaking. Then 1.5 mL samples of the cultures were harvested by adding RNAprotect bacterial reagent (Qiagen, Germany) to them followed by total RNA isolation using the RNeasy minikit (Qiagen, Germany) with on‐column DNA digestion by DNase I treatment, according to the manufacturer's instructions (Qiagen, Germany). The integrity of the RNA was validated by separating 1 μg of RNA on 8% denaturing acrylamide gels (7 M urea, 45 mM Tris base, 45 mM boric acid, 1 mM EDTA, 8% acrylamide‐bisacrylamide [19:1, weight/weight]), followed by staining of the gel with 0.5 μg/mL ethidium bromide and visual inspection of the 23S rRNA and 16S rRNA bands.

The preparation of RNA‐seq libraries was performed according to methods described previously (Borries et al. 2012), with minor adaptations. The synthesis of cDNA libraries was performed by the Cologne Center for Genomics (CCG) (http://portal.ccg.uni‐koeln.de/ccg/) using an Illumina TruSeq RNA library kit according to the manufacturer's instructions. Three biological replicates of each strain were sequenced, with 20 million reads each, using an Illumina HiSeq 4000 instrument. The read quality was assessed using MultiQC and the respective date were provided by the CCG. For data analysis, the sequencing raw data FASTQ files were uploaded to the public Galaxy Web platform (https://usegalaxy.org/) (Afgan et al. 2016). Sequencing reads were aligned to the *E. coli* K‐12 MG1655 genome (GenBank assembly accession number GCA_000005845.2) using Bowtie2 (Langmead and Salzberg 2012) with default settings for paired‐end libraries. These generated BAM files containing the aligned reads were used to calculate individual gene abundances using HTSeq (Anders et al. 2014) in union mode for stranded data sets with a minimum alignment quality of 10. Tabular lists of gene abundances for each sample were used for differential expression analysis using DEseq2 (Love et al. 2014) with default settings. The tabular lists were compiled and filtered using Excel (Microsoft). In addition, the aligned reads compiled in the BAM files of the individual samples were inspected using IGV (Integrative Genomics Viewer) (Robinson et al. 2011).

### 4.9 Identification of YdcI Binding Motif

The DNA sequence of the YdcI targets (Table S1) showing specific binding by YdcI as determined by EMSA was submitted to MEME‐suite (Bailey et al. 2015) to generate a motif.

## Author Contributions

**Karin Schnetz:** conceptualization, funding acquisition, writing – original draft, writing – review and editing, project administration, resources, supervision, data curation, validation, visualization, methodology. **Ria Basu Roy:** writing – original draft, investigation, methodology, visualization, formal analysis, validation, data curation.

## Funding

This work was supported by Deutsche Forschungsgemeinschaft (SCHN371/11‐1).

## Ethics Statement

The authors have nothing to report.

## Conflicts of Interest

The authors declare no conflicts of interest.

## Supporting information


**Figure S1:** Synthesis of the LTTRs LrhA, YafC, YdcI, and YhaJ determined by Western blotting.
**Figure S2:** Visualization of ChIP‐exo data for YdcI targets in IGV browser.
**Figure S3:** YdcI does not bind to its promoter regulatory DNA.
**Table S1:** Input sequence for defining a YdcI DNA‐binding loci using MEME‐suite.
**Table S2:** *E. coli* K12 strains.
**Table S3:** Plasmids.
**Table S4:** Oligonucleotides and DNA fragments.
**Table S5:** RNA‐seq data of LTTRs.

## Data Availability

The data that support the findings of this study are openly available in NCBI's Gene Expression Omnibus database at https://www.ncbi.nlm.nih.gov/geo/, reference number GSE304471. Data presented in this manuscript are included in the doctoral thesis by Ria Basu ROY that has been accepted by the Faculty of Mathematics and Natural Sciences of the University of Cologne, Germany, and deposited at the University of Cologne's library server at https://kups.ub.uni‐koeln.de/78875/.
