# Supplementary material for: Regulation by the Transcriptional Regulator YdcI in E. coli and (p)ppGpp‐Dependent Activation of ydcI Expression
Source: Mol Microbiol. 2026 Sep 11;126(5):503–18. doi: 10.1111/mmi.70110 (PMC13725319; doi:10.1111/mmi.70110)
Supplement: Supplementary file 1 [file MMI-126-503-s001.pdf]

Supplementary information to

Regulation by the transcriptional regulator YdcI in *E. coli* and (p)ppGpp-dependent activation of *ydcI* expression

Ria Basu ROY and Karin SCHNETZ*

Institute for Genetics, Department of Biology, Faculty of Mathematics and Natural Sciences, University of Cologne, Zülpicher Str, 47a, 50674 Cologne, Germany

corresponding author: schnetz@uni-koeln.de

The supplementary information includes
Figures S1 to S3
Table S1 Input DNA sequences for defining a YdcI DNA-binding sequence logo with MEME-suite
Table S2 Strains
Table S3 Plasmids
Table S4 Oligonucleotides
Table S5 RNA-seq data

Figures

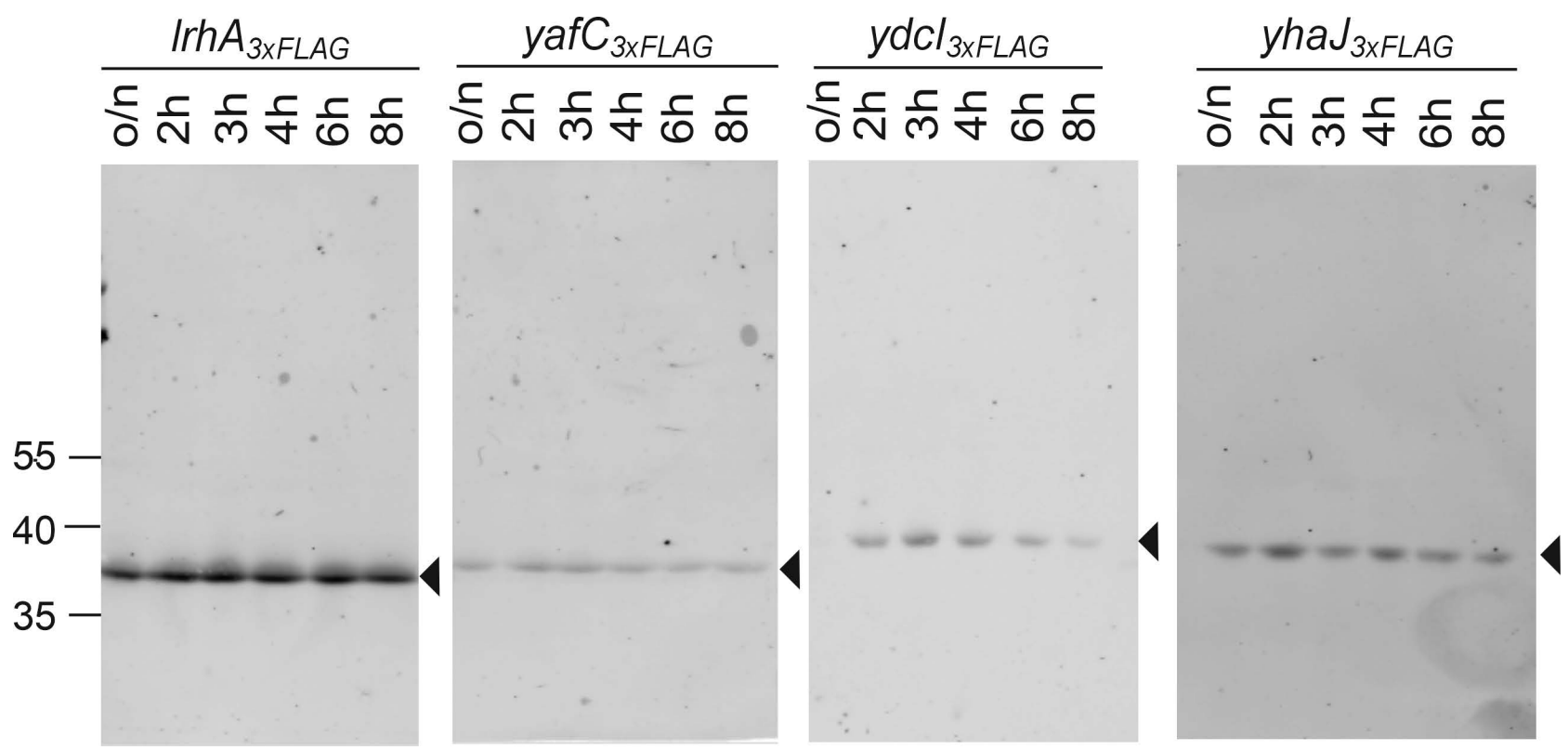

Figure S1. Synthesis of the LTTRs LrhA, YafC, YdcI, and YhaJ determined by Western blotting. Strains carrying 3xFLAG tagged alleles at their native locus encoding *lrhA*$_{3xFLAG}$ (U253), *ydcI*$_{3xFLAG}$ (U255), *yafC*$_{3xFLAG}$ (U254), and *yhaJ*$_{3xFLAG}$ (U263), respectively, were grown in tryptone medium and aliquots were harvested from over-night cultures (o/n) and after 2 to 8 hours, as indicated. Blots were incubated with Anti-FLAG® M2 (1:2000, Sigma F3165) as primary antibody and secondary Goat Anti-Mouse IgG H&L (Alexa Fluor® 680) antibody (1:10000). Imaging of protein bands was performed with Odyssey V3.0 software.

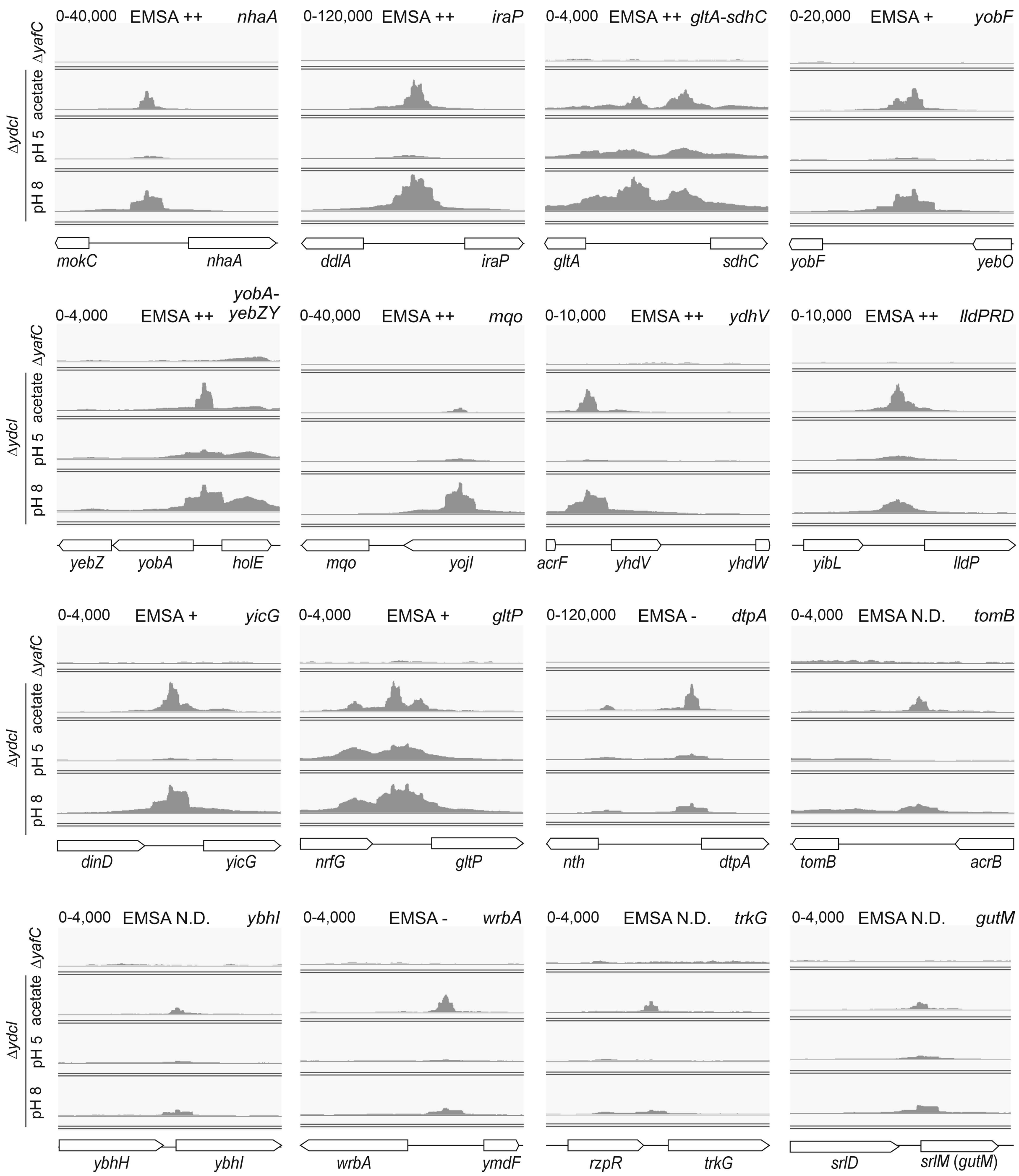

Figure S2 Visualisation of ChIP-exo data for YdcI targets in IGV browser. To visualize ChIP-exo data (Gao *et al.*, 2018) whole genome BAM files for *E. coli* K-12 *yafC-3xFLAG* (grown in acetate medium, and taken as control) and *ydcI-3xFLAG* (grown in acetate medium, and M9 minimal media pH 5 and pH 8, respectively) were used. The reads are shown as the grey graphs and are depicted for all the ChIP-exo identified targets of YdcI (Gao *et al.*, 2018). The data range of the reads is variable and depicted at the top of each graph, the window size of each graph is 1000 bp. The graphs are shown in the same order as the genes in Table 1. Also indicated is whether DNA-binding was observed in EMSA with the strength of DNA-binding specified as ++ (strong), + (weak), - (no specific binding), and N.D. (not determined).

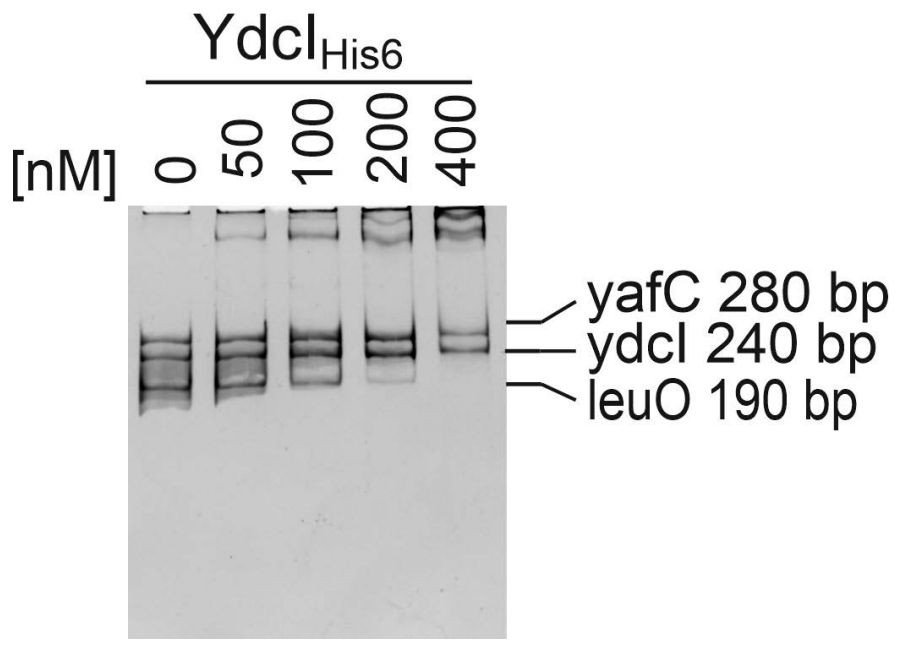

Figure S3 YdcI does not bind to its promoter regulatory DNA. Electrophoretic mobility shift assays were performed using a mixture of *yafC, ydcI, leuO* fragments incubated with increasing concentrations of purified YdcI$_{His6}$. Oligonucleotides used to amplify DNA fragments (length in bps shown next to the fragments) are listed in table S4. The samples were separated on 8% native polyacrylamide gels. Gel pictures are shown with inverted colors. DNA-binding reactions were set up on ice in 1X binding buffer (20mM Tris-HCl [pH 7.5], 100mM KCl, 2mM dithiothreitol [DTT] and 10% glycerol) with 10 nM DNA fragment for each DNA fragment in a reaction volume of 10 µl. The samples were incubated at 30°C for 20 min and then loaded onto an 8% polyacrylamide gel (acrylamide-bisacrylamide [29:1] in 0.5x TBE [45mM Tris base, 45mM boric acid, 1mM EDTA]). The gel was run for 10 min at 50 V, followed by 45 min at 200 V. The gel was stained with ethidium bromide for 20 min followed by imaging with GelDoc from Biorad company.

Tables

Table S1: Input sequence for defining a YdcI DNA-binding loci using MEME-suite		
Loci	Coordinates	Sequence
nhaA	17121-17330	TTTTTGTGGCGTAATTTTTTAATGATTTAATTATTTAACTTTAATTTATCTCTTCATCG<br>CAATTATTGACGACAAGCTGGATTATTTTTGAAATATTGGCCTAACAAGCATCGCCG<br>ACTGACAACAAATTAATTATTACTTTTCCTAATTAATCCCTCAGGAATCCTCACCTTA<br>AGCTATGATTATCTAGGCTTAGGGTCACTCGTGAGC
iraP	401098-401202	AGCTAATTTATTCCTGGCTTAAATGGCCATGCGGTGAGTTTTTTTCTCTTAATTATAA<br>GTTAACGAAGAGAATATATTTCATAACTTTTATTTATAATAAAGGTT
gltA-sdhCDAB	754588-754807	CTGGGTACAGAGCTCTGGGCGCTTGCAGGTAAAGGATCCATTGATGACGAATAAA<br>TGGCGAATCAAGTACTTAGCAATCCGAATTATTAAACTTGTCTACCACTAATAACTG<br>TCCCGAATGAATTGGTCAATACTTCCACACTGTTACATAAGTTAATCTTAGGTGAAA<br>TACCGACTTCATAACTTTTACGCATTATATGCTTTTCCTGGTAATGTTTGT
yobF	1907821-1908100	GTTGTTCCAGGCCAGTGGGAGAAGTTATTACATAGTGCGTGCAATATCACATTTTTT<br>GCTATGCAATGAATAAAAAGTTATATCACTTTTTCTCATAAAACAGTCAGTTAACGG<br>CTATTAATTACCCTAAAGAGAAGTCAATCCCCAAAGGGATTGTAAATTTAAAATAA<br>GAAAAATTGATGAATGAGCAAAAAAATCAAGAGAGAAACGTTTCGCTAATAAATA<br>ATACCACGCTTAATCACAAATCCGATGTAAACATCCTAATAAATTAATGGGGTAA
yobA-yebZY	1924941-1925150	TAGCGAAGGGAGCGTGCAGTTGAAGCCATATTATCTATTCCTTTTTGTAATAACTTT<br>TTTACAGAGCATAACCTTGTCTAATGTCTGAGTCGAGGATCATCAATTCCGGCTTGC<br>CATCCTGGCTCACTCTTAGTAACTTTTGCCCGCGAATGATGAGGAGATTAAGAATG<br>CTGAAGAATCTGGCTAAACTGGATCAAACAGAAATGGATA
mqo	2307145-2307424	CCTGATAAAACTCACGACGGAAGTGTGGATCCTGATCCGCCGCCCATTCATCCAGC<br>AGGATAATATCGCGTTCTTCTGCCAGCGCCAGCAACAGCGCCACGCGTTTTTTCTGC<br>CCTTTTGATAACTTCAGGTTAACAATACGCCCGTTGCTTAACTCAAGCTTATGAGCC<br>ATTTTCAGCTGCGCCAGCCACTTCTCAACCAGTTGCGGGTTAGCGGGTTTACCCTCC<br>GGCCCCAGCAGTTGATCAAACAGCCAGACATCGGTAAACACTGCCGAAAACAG
yhdV	3418135-3418394	TAAATCAGAAACATAAAGGCGCTTTCGGGTGCCTTTATTATTTCCAGTGAAACCCAT<br>AAAAATTAATAAGATATTCTTCTGCTCACTCTTTAAAAGCTTTCTATAGTTCCCGCTC<br>CCTTCACTATTTTTACAATTCACATAATTTGTCATTATTCATTCCGCAAGATTTATAAC<br>CTCCGGTGATAAAATGGCATTCAGCTCGTTAATAAGAGAGTTAACTTATTAAGCGT<br>TAGCGTTTATTACTGAGGTAACACCATGAA
lldPRD	3777164-3777363	CAGACATCTCCCCCCACAAGAATTGGCCCTACCAATTCTTCGCTTATCTGACCTCTG<br>GTTCACAATTTCCCAATTAAAACTCACATCAATGTTGCCAATACATAACATTTAGTTA<br>ACCATTCATTGTCATTATCCCTACACAACACAATTGGCAGTGCCACTTTTACACAAC<br>GTGTGACAAGGAGATGAGCAACAGACTC
yicG	3818587-3818866	GAAACTAAAAACTCTTTTTGTTGATTGAGACACCCGATGCGTAAGCCAAGGTCCAG<br>GTGCAGTGAACACAATGGCTAAATATTGCACCTTTCTTTTCCCCCTCAGTTTTAACCT<br>ATTTTTTCTTATGCATTTTCTCAGACAAGAAGTCAGAAGAATGCATCTCTGCTACAG<br>AAAATAGCGATTTCACATAACTACAAGTTATCAATTTCCCCTCCCCTTAAAAAATCTC<br>AATCGTGACAATGCGCACAAATCGCTACCCTGCCAGACAGATTTTTAGGGA
gltP	4294148-4294367	TGATGAAGATTATGTCATTGAGCTGCATAAAAAATAATCGAATGAACATATGCCAA<br>AAATAATCACTAATCAGTATTATTGCAGATTAAACAAATAAAAATCTTTCCATAACA<br>AATGGTTATTCATTAATCCTGCCATAAACTCGCGTTATTATGCATTAATGCAGCGAA<br>AAGCTCTGTTGTTAAAGGGTTGCGCAACATACCGCGCAAATGATACTGAT

**Table S2: *E. coli* K12 strains**		
Strains	Genotype / description[a]	Construction /reference[b]
CE41(DE3)	BL21(DE3) derivate for toxic protein expression	(Miroux & Walker, 1996)
M182	Δ(*lacIPOZYA)74 galU galK strA stpA::tet*<br>(lab storage number S159)	(Zhang *et al.*, 1996)
S3754	MG1655 Δ*hns*$_{\text{KD4-kan}}$	(Rangarajan & Schnetz, 2018)
T1241	BW30270 *ilvG*$^{+}$, *fhlDC*(IS1) highly motile, *dgcJ*::IS1	(Pannen *et al.*, 2016)
AB036	MG1655 Δ*relA*$_{\text{kan}}$ *spoT*$_{E319Q}$ zib563::Tn10, Lab number T2985	(Bougdour & Gottesman, 2007)
AB037	MG1655 *spoT*$_{E319Q}$ zib563::Tn10, Lab number T2986	(Bougdour & Gottesman, 2007)
JW5226-1	BW25113 Δ*ydcI783*$_{\text{kan}}$, Lab number T2034	(Baba *et al.*, 2006)
N4235	*rpoB*35* Δ*relA251*$_{\text{kan}}$ Δ*spoT207*$_{\text{cm,}}$ Lab number T1098	(Trautinger *et al.*, 2005)
JW0198-1	BW25113 Δ*yafC727*$_{\text{kan,}}$ Lab number T2791	(Baba *et al.*, 2006)
JW3076-1	BW25113 Δ*yhaJ724*$_{\text{kann,}}$ Lab number T2793	(Baba *et al.*, 2006)
U4	T1241 Δ*lacZ*	(Yilmaz *et al.*, 2020)
U78	T1241 Δ*ara* Δ*lac* Δ*lrhA*$_{\text{KD3-cm}}$	(Breddermann & Schnetz, 2017)
U253	T1241 *lrhA*$_{3XFLAG\ \text{kan}}$	T1241/pKD46 x OA816/OA817 (pSUB11)
U254	T1241 *yafC*$_{3XFLAG\ \text{kan}}$	T1241/pKD46 x OA826/OA827 (pSUB11)
U255	T1241 *ydcI-3xFLAG*$_{\text{kan}}$	T1241/pKD46 x OA824/OA825 (pSUB11)
U261	U4 Δ*hns*$_{\text{FRT}}$	U4 x P1*vir*(S3754 Δ*hns*$_{kan}$), followed by flipping using pCP20
U263	T1241 *yhaJ*$_{3XFLAGF\ \text{kan}}$	T1241/pKD46 x OA822/OA823 (pSUB11)
U451	U4 Δ*yafC*$_{\text{kan}}$	U4 x P1vir [JW0198-1 Δ*yafC*$_{\text{kan}}$]
U453	U4 Δ*yhaJ*$_{\text{kan}}$	U4 x P1vir [JW3076-1 Δ*yhaJ* $_{\text{kan}}$]
U456	U4 Δ*yafC*$_{\text{FRT}}$	U451 x pCP20
U458	U4 Δ*yhaJ*$_{\text{FRT}}$	U453 x pCP20
U502	U4 Δ*lrhA*$_{\text{cm}}$	U4 x P1vir [U78 Δ*lrhA*$_{\text{KD3-cm}}$]
U503	U4 Δ*ydcI*$_{\text{kan}}$	U4 x P1vir [JW5226-1 Δ*ydcI*$_{\text{kan}}$]
U504	U4 Δ*lrhA*$_{\text{FRT}}$	U502 x pCP20
U505	U4 Δ*ydcI*$_{\text{FRT}}$	U503 x pCP20
U556	U505 Δ*hns*$_{\text{kan}}$	U505 x P1vir [S3754 Δ*hns*$_{\text{KD4-kan}}$]
U558	U505 Δ*hns*$_{\text{FRT}}$	U556 x pCP20
U559	U4 Δ*stpA::tet*	U4 x P1vir [M182 *stpA::tet*]
U560	U505 Δ*stpA::tet*	U505 x P1vir [M182 *stpA::tet*]
U561	U4 Δ*stpA::tet* Δ*hns*$_{\text{kan}}$	U559 x P1vir [S3754 Δ*hns*$_{\text{KD4-kan}}$]
U562	U505 Δ*stpA::tet* Δ*hns*$_{\text{kan}}$	U560 x P1vir [S3754 Δ*hns*$_{\text{KD4-kan}}$]
U563	U4 Δ*stpA::tet* Δ*hns*$_{FRT}$	U561 x pCP20
U564	U505 Δ*stpA::tet* Δ*hns*$_{FRT}$	U562 x pCP20
U571	U4 Δ*relA*$_{kan}$	U4 x P1vir [N4235 Δ*relA*$_{\text{kan}}$ Δ*spoT*$_{\text{cm}}$]
U572	U505 Δ*relA*$_{kan}$	U505 x P1vir [N4235 Δ*relA*$_{\text{kan}}$ Δ*spoT*$_{\text{cm}}$]
U577	U4 *ydcI-3xFLAG*$_{\text{kan}}$	U4 x P1vir [U255 *ydcI-3xFLAG*$_{\text{kan}}$]
U578	U4 Δ*relA*$_{kan}$ Δ*spoT*$_{\text{cm}}$	U571 x P1 vir [N4235 Δ*relA*$_{\text{kan}}$ Δ*spoT*$_{\text{cm}}$]
U579	U505 Δ*relA*$_{kan}$ Δ*spoT*$_{cm}$	U572 x P1 vir [N4235 Δ*relA*$_{\text{kan}}$ Δ*spoT*$_{\text{cm}}$]
U584	U4 *ydcI-3xFLAG*$_{\text{FRT}}$	U577 x pCP20
U613	U4 *spoT*$_{E319Q}$ zib563::Tn10	U4 x P1vir [AB037 *spoT*$_{E319Q}$ zib563::Tn10]
U614	U505 *spoT*$_{E319Q}$ zib563::Tn10	U505 x P1vir [AB037 *spoT*$_{E319Q}$ zib563::Tn10]
U615	*U4* Δ*relA*$_{\text{kan}}$ *spoT*$_{E319Q}$ zib563::Tn10	U571 x P1vir [AB037 *spoT*$_{E319Q}$ zib563::Tn10]
U616	U505 Δ*relA*$_{\text{kan}}$ *spoT*$_{E319Q}$ zib563::Tn10	U572 x P1vir [AB037 *spoT*$_{E319Q}$ zib563::Tn10]

**Table S2: *E. coli* K12 strains**		
Strains	Genotype / description[a]	Construction /reference[b]
U621	T1241 Δ*phoB*-Δ*phoR*$_{kan}$	T1241/pKD46 x OB483/OB484 (pKD13)
U622	U4 Δ(*phoB*-phoR)$_{kan}$	U4 x P1vir [U621 Δ*phoB*-Δ*phoR*$_{kan}$]
U623	U505 Δ(*phoB*-phoR)$_{kan}$	U505 x P1vir [U621 Δ*phoB*-Δ*phoR*$_{kan}$]
U624	U4 Δ(*phoB*-phoR)$_{FRT}$	U622 x pCP20
U625	U505 Δ(*phoB*-phoR)$_{FRT}$	U623 x pCP20

[a]Abbreviations and genetic features are as follow: kanamycin resistance (kan); chloramphenicol resistance (cm); FLP recombinase target site (FRT).

[b] Strains which were constructed by transduction is stated as “x phage [donor strain]”; λ Red recombineering, stated as “x PCR primer pair (template)” followed by Flp recombinase-catalyzed deletion of the resistance marker, “x pCP20”.

**Table S3: Plasmids**		
Plasmid	Features*	Reference
pCP20	*cI*$_{857}$ P_R *flp* in pSC101-ori rep^{ts} Amp	(Cherepanov & Wackernagel, 1995)
pKD4	FRT-*kan*-FRT oriRγ Amp	(Datsenko & Wanner, 2000)
pKD46	*araC Para* γ-*β-exo* in pSC101-ori rep^{ts} Amp	(Datsenko & Wanner, 2000)
pET22b(+)	P_{T7} MCS His6 in pMB1-ori Amp	Novagen®
pSUB11	*3xFLAG* in pKD4	(Uzzau *et al.*, 2001)
pKESL317	*ydcI*$_{His6}$ (full-length) in pET-22b(+)	PCR OA865/OA866 of T1241 in pET22b(+) (NdeI, XhoI)
pKETS24	*lacI*^{q} P_{UV5} MCS in pSC-ori Cm	(Fragel *et al.*, 2019)
pKECY26	*mNeonGreen* in pSC-ori Cm	mNeonGreen fragment in pKETS24
pKECY49	MCS *lacZ* in pSC-ori Cm	MCS (OA899/OA900) *lacZ* in pKECY26
pKERBR91	P_{iraP} *lacZ* in pSC-ori Cm	P_{iraP} (PCR OB310/OB311) in pKECY49
pKERBR104	P_{phoA} *lacZ* in pSC-ori Cm	P_{phoA} (PCR OB310/OB313) in pKECY49
pKERBR93	P_{ydcI} *lacZ* in pSC-ori Cm	P_{ydcI} (PCR OB314/OB315) in pKECY49
pKERBR114	P_{ydcI} *lacZ* in pSC-ori Tet	*tetR* (PCR OB601/OB602) from pSC101 in pKERBR93
pKERBR115	P_{iraP} *lacZ* in pSC-ori Tet	*tetR* (PCR OB601/OB602) from pSC101 in pKERBR91
pKERBR90	P_{lldPRD} *lacZ* in pSC-ori Cm	P_{lldP} (PCR OB308/OB309) in pKECY49
pKERBR89	$P_{yobA\text{-}yebZY}$ *lacZ* in pSC-ori Cm	P_{yobA} (PCR OB306/OB307) in pKECY49
pKERBR113	P_{mqo} *lacZ* in pSC-ori Cm	P_{mqo} (PCR OB499/OB500) in pKECY49
pKERBR112	P_{yhdV} *lacZ* in pSC-ori Cm	P_{yhdV} (PCR OB485/OB486) in pKECY49
pKERBR111	P_{gltA} *lacZ* in pSC-ori Cm	P_{gltA} (PCR OB422/OB423) in pKECY49
pKERBR105	$P_{sdhCDAB}$ *lacZ* in pSC-ori Cm	P_{sdhC} (PCR OB405/OB406) in pKECY49
pKERBR122	P_{nhaA} *lacZ* in pSC-ori Cm	P_{nhaA} (PCR OB700/OB701) in pKECY49
pKERBR123	P_{gltP} *lacZ* in pSC-ori Cm	P_{gltP} (PCR OB702/OB703) in pKECY49

Abbreviations and features are as follow: chloramphenicol resistance (Cm); tetracycline resistance (Tet); ampicillin resistance (Amp); MCS for multiple cloning site; rep^{ts} for temperature sensitive plasmid replication; His6 for 6x histidine tag; 3x FLAG for DYKDHDGDYKDHDIDYKDDDDK (Sigma-Aldrich, Germany).

**Table S4: Oligonucleotides and DNA fragments**		
Number	Sequence*	Description
OA824	CCAGTTGATGATTAACGCTATTCGAAAATCAATGCCGTTC gactacaaagaccatgacggtgattata	construction of *ydcI-3xFLAG*$_{kan}$
OA825	AAATGTTCAAGGAGGGATCGACAGATCCCTTCACCTTcatatga atatcctccttagttcctattcc	construction of *ydcI-3xFLAG*$_{kan}$
OB483	GACGTCGCATTAATGATCGCAACCTATTTATTACAACAGGG CAAATCATGattccggggatccgtcgacc	construction of Δ(*phoB*-phoR)$_{kan}$
OB484	TTATGGCAATAAAAGATGACAAAGGCGGATTAATCGCTGTTT TTGGCAATtgtaggctggagctgcttcga	construction of Δ(*phoB*-phoR)$_{kan}$
OA865	tcaccatATGGAAAAAAATAGTCTGTTTAGTCAGCG	Cloning *ydcI*$_{His6}$ under P$_{tac}$
OA866	tcacctcgagGAACGGCATTGATTTTCGAATAGCG	Cloning *ydcI*$_{His6}$ under P$_{tac}$
OA899	5'P-TCGACATCTGCAGACT	Cloning MCS in pKECY26
OA900	5'P-CTAGAGTCTGCAGATG	Cloning MCS in pKECY26
OB310	gcaggtcgacTGATTTACCACCAAAAACGATTCCTA	*iraP, phoA* promoter *lacZ* fusion
OB311	gcagtctagaAAATAACAACTCAGCAATGAGATTTTTCAT	*iraP* promoter *lacZ* fusion
OB313	gcagtctagaGAGTGCCAGTGCAATAGTGCTTTG	*phoA* promoter *lacZ* fusion
OB314	gcagtctagaGCGCTGACTAAACAGACTATTTTTTTCC	*ydcI* promoter *lacZ* fusion
OB315	gcaggtcgacAATCTCATCCGCCGTGATGCT	*ydcI* promoter *lacZ* fusion
OB601	gcagagatctTTCTCATGTTTGACAGCTTATCATCG	*tetR* insertion in pKERBR91 and pKERBR93
OB602	gcagggcgcgccATGGCGGACGCGATGGA	*tetR* insertion in pKERBR91 and pKERBR93
OB306	gcagtctagaGTAGCGAAGGGAGCGTGCAGT	*yobA-yebZY* promoter *lacZ* fusion
OB307	gcaggtcgacTGTTTGATCCAGTTTAGCCAGATTCTT	*yobA-yebZY* promoter *lacZ* fusion
OB308	gcaggtcgacCAGGAGATGACTGGGTTTTCAAAG	*lldPRD* promoter *lacZ* fusion
OB309	gcagtctagaGGGATCGTAGTTTTGTTGCCAGAGAT	*lldPRD* promoter *lacZ* fusion
OB499	gcagtctagaCGAGAAGAGCATGGCAGTCACTTT	*mqo* promoter *lacZ* fusion
OB500	gcaggtcgacTGCTGTTTCTGATTGGCGGC	*mqo* promoter *lacZ* fusion
OB485	gcaggtcgacTAAATCAGAAACATAAAGGCGCTTTC	*yhdV* promoter *lacZ* fusion
OB486	gcagtctagaAGCCAGTGTGGCTATCATCATCTT	*yhdV* promoter *lacZ* fusion
OB422	gcaggtcgacAGGTCTTTGTTTTTTCACATTTCTTATCAT	*gltA* promoter *lacZ* fusion
OB423	gcagtctagaGAGGGTGAGTTTTGCTTTTGTATCAG	*gltA* promoter *lacZ* fusion
OB405	GCAGgtcgacGAGGGTGAGTTTTGCTTTTGTATCAG	*sdh* promoter *lacZ* fusion
OB406	gcagtctagaAGGTCTTTGTTTTTTCACATTTCTTATCAT	*sdh* promoter *lacZ* fusion
OB700	gcaggtcgacAGTAAGAGGCACTCTACATGTGTTCAG	*nhaA* promoter *lacZ* fusion
OB701	gcagtctagaACTGCTAAAGAATCGATGCAGATGTTTC	*nhaA* promoter *lacZ* fusion
OB702	gcaggtcgacGCGAAATTGTTGCAGCGGAGATTGGA	*gltP* promoter *lacZ* fusion
OB703	gcagtctagaCCAGGCCAGGCTGAATTTTATATTTTT	*gltP* promoter *lacZ* fusion
OB324	6-FAM-CGGGGGATTCGCGGCCTT	*iraP* EMSA fragment A
OB325	CGGGGGATTCGCGGCCTT	*iraP* EMSA fragment A
OB326	TTCTCTTCGTTAACTTATAATTAAGAGAAAAAAAC	*iraP* EMSA fragment A
OB327	6-FAM-AGCTAATTTATTCCTGGCTTAAATGGC	*iraP* EMSA frag. B, /B1, B4, mut1, mut2, mut1/2
OB328	AGCTAATTTATTCCTGGCTTAAATGGC	*iraP* EMSA frag. B, /B1, B4, mut1, mut2, mut1/2
OB329	ACTTTGCGCAAATATTTTTTGTTTGATTACCA	*iraP* EMSA fragment B, B3
OB330	6-FAM-TTTTGTGTGGGTAATTATTTAAATAAGAGAAAC	*iraP* EMSA fragment C
OB331	TTTTGTGTGGGTAATTATTTAAATAAGAGAAAC	*iraP* EMSA fragment C
OB332	AAATAACAACTCAGCAATGAGATTTTTCAT	*iraP* EMSA fragment C
OB334	AACCTTTATTATAAATAAAAGTTATGAAATATATTCTCT	*iraP* EMSA fragment B1
OB335	6-FAM-CTCTTAATTATAAGTTAACGAAGAGAATATATTTCA	*iraP* EMSA fragment B2
OB336	CTCTTAATTATAAGTTAACGAAGAGAATATATTTCA	*iraP* EMSA fragment B2

**Table S4: Oligonucleotides and DNA fragments**		
Number	Sequence*	Description
OB337	CTTATTTAAATAATTACCCACACAAAATATAGGC	*iraP* EMSA fragment B2
OB338	6-FAM-TTTATAATAAAGGTTGATAATTAAAAGCCTATATTT	*iraP* EMSA fragment B3
OB339	TTTATAATAAAGGTTGATAATTAAAAGCCTATATTT	*iraP* EMSA fragment B3
OB618	CACAAAATATAGGCTTTTAATTATCAACCTTTATTA	*iraP* EMSA fragment B4, mut1, mut2, mut1/2
OB321	6-FAM-TAGCGAAGGGAGCGTGCAGT	*yobA-yebZY* EMSA fragment Y
OB340	TAGCGAAGGGAGCGTGCAGT	*yobA-yebZY* EMSA fragment Y
OB307	GCAG<u>gtcgac</u>TGTTTGATCCAGTTTAGCCAGATTCTT	*yobAyebZY* EMSA fragment Y
OB343	6-FAM-CTGGGTACAGAGCTCTGGGCG	*gltA* EMSA fragment A1
OB344	CTGGGTACAGAGCTCTGGGCG	*gltA* EMSA fragment A1
OB345	ACAAACATTACCAGGAAAAGCATATAATG	*gltA* EMSA fragment A1, A2
OB616	6-FAM-CGAATGAATTGGTCAATACTTCCACA	*gltA* EMSA fragment A2
OB617	CGAATGAATTGGTCAATACTTCCACA	*gltA* EMSA fragment A2
OB346	6-FAM-TAGGTGAAATACCGACTTCATAACTTTTAC	*gltA* EMSA fragment A3
OB347	TAGGTGAAATACCGACTTCATAACTTTTAC	*gltA* EMSA fragment A3
OB348	GTTCCGGAGACCTGGCGG	*gltA* EMSA fragment A3
OB501	6-FAM-CGAGAAGAGCATGGCAGTCACTT	*mqo* EMSA fragment M1
OB502	CGAGAAGAGCATGGCAGTCACTT	*mqo* EMSA fragment M1
OB503	CCGGACGGCATAATATTACGAC	*mqo* EMSA fragment M1
OB504	6-FAM-AGCATATTGACCTGACGGCAGC	*mqo* EMSA fragment M2
OB505	AGCATATTGACCTGACGGCAGC	*mqo* EMSA fragment M2
OB506	TCCTGCTGGATGAATGGGC	*mqo* EMSA fragment M2
OB507	6-FAM-CCTGATAAAACTCACGACGGAAGTG	*mqo* EMSA fragment M3
OB508	CCTGATAAAACTCACGACGGAAGTG	*mqo* EMSA fragment M3
OB509	CTGTTTTCGGCAGTGTTTACCGAT	*mqo* EMSA fragment M3
OB510	6-FAM-ACCCTCCGGCCCCAGC	*mqo* EMSA fragment M4
OB511	ACCCTCCGGCCCCAGC	*mqo* EMSA fragment M4
OB512	TGCTGTTTCTGATTGGCGGC	*mqo* EMSA fragment M4
OB487	6-FAM-TAAATCAGAAACATAAAGGCGCTTTC	*yhdV* EMSA fragment V1
OB488	TAAATCAGAAACATAAAGGCGCTTTC	*yhdV* EMSA fragment V1
OB489	TTCATGGTGTTACCTCAGTAATAAACGC	*yhdV* EMSA fragment V1
OB490	6-FAM-CGTTAATAAGAGAGTTAACTTATTAAGCGTTAGC	*yhdV* EMSA fragment V2
OB491	CGTTAATAAGAGAGTTAACTTATTAAGCGTTAGC	*yhdV* EMSA fragment V2
OB492	GGCAATAGCCCCAGCCGT	*yhdV* EMSA fragment V2
OB493	6-FAM-ACCAATACCTGTATGAAAGGCACGA	*yhdV* EMSA fragment V3
OB494	ACCAATACCTGTATGAAAGGCACGA	*yhdV* EMSA fragment V3
OB495	CGAAATAACGCGATGTAACATGC	*yhdV* EMSA fragment V3
OB496	6-FAM-ATTGCTATATTCTGCTGGGTAATTCCC	*yhdV* EMSA fragment V4
OB497	ATTGCTATATTCTGCTGGGTAATTCCC	*yhdV* EMSA fragment V4
OB498	CTGAGTGTAGACAGGAAATAATAAAAACAGC	*yhdV* EMSA fragment V4
OB349	6-FAM-CAGACATCTCCCCCCACAAGA	*lldPRD* EMSA fragment L
OB350	CAGACATCTCCCCCCACAAGA	*lldPRD* EMSA fragment L
OB351	GAGTCTGTTGCTCATCTCCTTGTCAC	*lldPRD* EMSA fragment L
OB515	6-FAM-ATAGGAGGCCTCGGGTTGATG	*nhaA* EMSA fragment N1
OB516	GCCAATATTTCAAAAATAATCCAGCTT	*nhaA* EMSA fragment N1
OB517	ATAGGAGGCCTCGGGTTGATG	*nhaA* EMSA fragment N1
OB663	6-FAM-TTTTTGTGGCGTAATTTTTTAATGATTTAA	*nhaA* EMSA fragment N2
OB664	GCTCACGAGTGACCCTAAG	*nhaA* EMSA fragment N2
OB665	TTTTTGTGGCGTAATTTTTTAATGATTTAA	*nhaA* EMSA fragment N2
OB518	6-FAM-TTAATTTATCTCTTCATCGCAATTATTGAC	*nhaA* EMSA fragment N3
OB519	CAGATTATAGTGTAGGAATAATTCGTTTTTTAC	*nhaA* EMSA fragment N3

Table S4: Oligonucleotides and DNA fragments		
Number	Sequence*	Description
OB520	TTAATTTATCTCTTCATCGCAATTATTGAC	*nhaA* EMSA fragment N3
OB521	6-FAM-CTGATGGCGCAAATTCTTCAATAG	*nhaA* EMSA fragment N4
OB522	CAGGATAGCGGCAATGATAAGAATAA	*nhaA* EMSA fragment N4
OB523	CTGATGGCGCAAATTCTTCAATAG	*nhaA* EMSA fragment N4
OB530	6-FAM-GCCCCGCTGTGGCTCTTG	*dtpA* EMSA fragment D1
OB531	CGACAGCATCGTGATCTTGATCAC	*dtpA* EMSA fragment D1
OB532	GCCCCGCTGTGGCTCTTG	*dtpA* EMSA fragment D1
OB533	6-FAM-AATTGTTTTTTTAATAGCGAAATTTTCTATTC	*dtpA* EMSA fragment D2
OB534	ATCCAGCGTTAAATTTTTCAAAAAAC	*dtpA* EMSA fragment D2
OB535	AATTGTTTTTTTAATAGCGAAATTTTCTATTC	*dtpA* EMSA fragment D2
OB536	6-FAM-CCCCTCTCAGTGCAGTGAAAAAA	*dtpA* EMSA fragment D3
OB537	GTTGGTTTTTGGTTTGCAGTGGA	*dtpA* EMSA fragment D3
OB538	CCCCTCTCAGTGCAGTGAAAAAA	*dtpA* EMSA fragment D3
OB539	6-FAM-TGTACCTCGTTTAACCCGAAATCTGT	*yobF* EMSA fragment F1
OB540	AGCAAAAAAATGTGATATTGCACGC	*yobF* EMSA fragment F1
OB541	TGTACCTCGTTTAACCCGAAATCTGT	*yobF* EMSA fragment F1
OB542	6-FAM-GTTGTTCCAGGCCAGTGGGA	*yobF* EMSA fragment F2
OB543	TTACCCCATTAATTTATTAGGATGTTTACATC	*yobF* EMSA fragment F2
OB544	GTTGTTCCAGGCCAGTGGGA	*yobF* EMSA fragment F2
OB545	6-FAM-AAATAATACCACGCTTAATCACAAATCC	*yobF* EMSA fragment F3
OB546	TGAAGACGAAGATATTATTCGTCTGGT	*yobF* EMSA fragment F3
OB547	AAATAATACCACGCTTAATCACAAATCC	*yobF* EMSA fragment F3
OB548	6-FAM-GAAACTAAAAACTCTTTTTGTTGATTGAGACA	*yicG* EMSA fragment G1
OB549	TCCCTAAAAATCTGTCTGGCAGG	*yicG* EMSA fragment G1
OB550	GAAACTAAAAACTCTTTTTGTTGATTGAGACA	*yicG* EMSA fragment G1
OB551	6-FAM-TGAAATCACCGCCCTGATGC	*gltP* EMSA fragment P1
OB552	ATTTTTGGCATATGTTCATTCGATTAT	*gltP* EMSA fragment P1
OB553	TGAAATCACCGCCCTGATGC	*gltP* EMSA fragment P1
OB554	6-FAM-TGATGAAGATTATGTCATTGAGCTGC	*gltP* EMSA fragment P2
OB555	ATCAGTATCATTTGCGCGGTATGT	*gltP* EMSA fragment P2
OB556	TGATGAAGATTATGTCATTGAGCTGC	*gltP* EMSA fragment P2
OB557	6-FAM-ATGCAGCGAAAAGCTCTGTTGTTA	*gltP* EMSA fragment P3
OB558	GGCCAGGCTGAATTTTATATTTTTCA	*gltP* EMSA fragment P3
OB559	ATGCAGCGAAAAGCTCTGTTGTTA	*gltP* EMSA fragment P3
OA103	ATAGTGTCTGCCTCCAGTGGTCAG	EMSA *ydcI* promoter region
OA999	TAAACAGACTATTTTTTTCCATAAGCGAT	EMSA *ydcI* promoter region
OA840	GCATGGCATAGGTGTTTTTTCG	EMSA *yafC* promoter region
OB001	CGACGTGGCTTTCATTTTTGCT	EMSA *yafC* promoter region
OA267	TTTGTATTGAATTATATGTTTTGCGATTTTT	EMSA *leuO* promoter region
OA268	TAATGTTTTTGTTGCGAAAACAATCTAA	EMSA *leuO* promoter region
Double stranded fragments		
M1	AGCTAATTTATTCCTGGCTTAAATGGCCATGCGGTGAGTTTTTTTCTCT**CGG**TTATAAG**CCG**ACGAAGAGAATATATTTCATAACTTTTATTTATAATAAAGGTTGATAATTAAAAGCCTATATTTTGTGGCCCG	mutation mut1 of YdcI site I at *iraP*
M2	AGCTAATTTATTCCTGGCTTAAATGGCCATGCGGTGAGTTTTTTTCTCTTAATTATAAGTTAACGAAGAGAATATATTTCA**CGG**CTTTTAT**CCG**TAATAAAGGTTGATAATTAAAAGCCTATATTTTGTG	mutation mut2 of YdcI site I at *iraP*

Table S4: Oligonucleotides and DNA fragments		
Number	Sequence*	Description
M1-M2	AGCTAATTTATTCCTGGCTTAAATGGCCATGCGGTGAGTTTTTTTCTCT**CGG**TTATAAG**CCG**ACGAAGAGAATATATTTCA**CGG**CTTTTAT**CCG**TAATAAAGGTTGATAATTAAAAGCCTATATTTGTG	mutation mut1/2 of YdcI site I at *iraP*

Oligonucleotide sequences are given in 5ˈ to 3ˈ direction. Sequences homologous to the template are shown in capital letters and the sites for restriction endonucleases are underlined. The mutations of YdcI binding sites are shown in bold and underlined.

Table S5: RNA-seq data of LTTRs

Gene	pos 1	pos 2	Function	WT/ΔlrhA log2 (FC)	P-adj	WT/ΔyafC log2 (FC)	P-adj	WT/ΔydcI log2 (FC)	P-adj	WT/ΔyhaJ log2 (FC)	P-adj
nhaA	17489	18655	Na+:H+ antiporter NhaA	-0.38	0.120	-0.32	0.128	-0.19	0.614	-0.35	0.243
carA	29651	30799	carbamoyl phosphate synthetase subunit α	1.72	0.000	2.34	0.000	1.35	0.006	1.09	0.034
rrlH	225759	228662	23S ribosomal RNA	1.00	0.000	1.63	0.000	0.86	0.053	0.72	0.177
dkgB	229167	229970	methylglyoxal reductase DkgB	0.33	0.380	2.44	0.000	0.05	0.937	0.44	0.258
gpt	255977	256435	xanthine-guanine phsophoribosyltransferase	1.43	0.000	1.68	0.000	0.77	0.174	1.45	0.000
codB	354922	356181	cytosine transporter	1.54	0.000	2.00	0.000	1.08	0.030	1.16	0.014
ddlA	399829	400923	D-alanine-D-alanine ligase A	-0.09	0.776	0.02	0.951	0.11	0.777	-0.06	0.924
iraP	401386	401646	anti-adaptor protein IraP	-0.27	0.509	-0.36	0.266	2.58	0.000	-0.06	0.929
phoA	401747	403162	alkaline phosphatase	-0.48	0.013	-0.55	0.001	2.07	0.000	-0.42	0.100
tomB	480334	480708	Hha toxicity modulator TomB	-0.91	0.000	-0.64	0.000	-0.35	0.468	-0.58	0.088
purK	551527	552594	5-(carboxyamino)imidazole ribonucleotide synthase	1.42	0.002	2.12	0.000	0.20	0.791	0.99	0.105
purE	552591	553100	N5-carboxyaminoimidazole ribonucleotide mutase	1.84	0.000	2.33	0.000	0.18	NA	1.77	0.000
borD	578600	578893	DLP12 prophage; prophage lipoprotein BorD	4.92	0.000	1.47	0.000	0.32	0.632	0.91	0.059
dcuC	654583	655968	anaerobic C4-dicarboxylate transporter DcuC	-3.29	0.000	-3.48	0.000	-1.21	0.031	-2.44	0.000
glnU	696865	696939	tRNA-Gln (UUG)	1.10	0.003	1.72	0.000	0.96	0.032	0.68	0.199
leuW	696963	697047	tRNA-Leu (UAG)	1.31	0.001	1.75	0.000	1.19	0.009	0.96	0.065
abrB	746723	747769	putative regulator	-1.62	0.000	-1.20	0.000	-0.99	0.042	-1.23	0.001
gltA	753185	754468	citrate synthase	-0.06	0.914	-0.44	0.028	-0.65	0.052	-0.09	0.859
sdhC	755177	755566	succinate:quinone oxidoreductase, membrane protein SdhC	0.82	0.003	0.44	0.020	0.10	0.827	0.43	0.309
sdhD	755560	755907	succinate:quinone oxidoreductase, membrane protein SdhD	0.58	0.077	0.27	0.363	-0.09	0.842	0.29	0.600
sdhA	755907	757673	succinate:quinone oxidoreductase, FAD binding protein	0.33	0.313	0.13	0.721	-0.21	0.523	0.20	0.703
sdhB	757689	758405	succinate:quinone oxidoreductase, iron-sulfur cluster binding protein	0.19	0.578	0.02	0.956	-0.31	0.293	0.08	0.893
valT	780765	780840	tRNA-Val	1.16	0.000	1.74	0.000	1.21	0.000	0.88	0.039
valZ	781068	781143	tRNA-Val	1.03	0.000	1.52	0.000	1.26	0.000	0.88	0.025
lysY	781147	781222	tRNA-Lys (UUU)	1.22	0.000	1.70	0.000	1.48	0.000	0.85	0.062
lysZ	781369	781444	tRNA-Lys (UUU)	1.15	0.000	1.72	0.000	1.37	0.000	0.91	0.032
lysQ	781577	781652	tRNA-Lys (UUU)	0.97	0.005	1.70	0.000	1.43	0.000	0.86	0.021
ybhI	801887	803320	putative tricarboxylate transporter	0.54	0.348	0.35	0.599	0.08	0.920	0.56	0.372
bioF	810381	811535	8-amino-7-oxononanoate synthase	-1.63	0.000	-1.55	0.000	-1.08	0.039	-1.46	0.001
bioC	811522	812277	malonyl-acyl carrier protein methyltransferase	-1.61	0.000	-1.59	0.000	-1.06	0.045	-1.44	0.000
bioD	812270	812947	dethiobiotin synthetase	-1.62	0.000	-1.73	0.000	-1.04	0.050	-1.50	0.000
ybiB	835248	836210	nonspecific DNA-binding protein YbiB	-0.36	0.148	-0.57	0.004	-0.44	0.201	-0.21	0.642
bssR	878248	878631	regulator of biofilm formation	-3.14	0.000	-3.35	0.000	-1.20	0.032	-2.50	0.000
hcp	912162	913814	protein S-nitrosylase	-1.41	0.000	-1.86	0.000	-1.16	0.023	-1.10	0.018
dmsA	940959	943403	dimethyl sulfoxide reductase subunit A	-2.27	0.000	-2.92	0.000	-1.19	0.039	-1.78	0.000
dmsB	943414	944031	dimethyl sulfoxide reductase subunit B	-1.56	0.000	-2.16	0.000	-1.13	0.036	-1.03	0.051
ycbJ	971752	972645	putative phosphotransferase YcbJ	-2.26	0.000	-2.49	0.000	-1.55	0.002	-1.76	0.000
wrbA	1067112	1067708	NAD(P)H:quinone oxidoreductase	-0.19	0.693	-0.03	0.948	0.48	0.343	0.53	0.311
ymdF	1068081	1068254	stress-induced bacterial acidophilic repeat motifs-containing protein YmdF	-0.46	0.150	0.53	0.055	0.87	0.003	0.82	0.003
ymdG	1079120	1079146	protein YmdG	0.65	0.338	1.91	0.001	1.21	NA	0.99	NA

Table S5: RNA-seq data of LTTRs

Gene	pos 1	pos 2	Function	WT/ΔlrhA log2 (FC)	P-adj	WT/ΔyafC log2 (FC)	P-adj	WT/ΔydcI log2 (FC)	P-adj	WT/ΔyhaJ log2 (FC)	P-adj
putP	1079305	1080813	proline:Na+ symporter	0.77	0.227	2.42	0.000	2.13	0.000	0.91	0.102
pgaA	1089866	1092289	partially deacetylated poly-beta-1,6-N-acetyl-D-glucosamine outer membrane porin	-1.55	0.000	-0.95	0.000	-0.66	0.086	-1.09	0.000
trhO	1116807	1117859	tRNA U34 hydroxylase TrhO	1.54	0.000	1.71	0.000	0.85	0.083	0.93	0.036
ychH	1258791	1259069	stress-induced protein	-1.73	0.000	-1.57	0.000	-1.17	0.015	-1.06	0.003
narK	1277957	1279348	nitrate:nitrite antiporter NarK	-1.67	0.002	-1.97	0.001	-0.81	0.159	-1.09	0.067
narG	1279864	1283607	nitrate reductase A subunit α	-1.77	0.001	-2.43	0.000	-0.95	0.100	-1.05	0.080
narH	1283604	1285142	nitrate reductase A subunit beta	-1.18	0.043	-1.70	0.002	-1.13	0.039	-0.84	0.136
tyrV	1287244	1287328	tRNA-Tyr (GUA)	1.30	0.001	1.56	0.000	1.23	0.004	0.87	0.088
ompW	1314020	1314658	outer membrane protein W	-1.51	0.000	-1.96	0.000	-1.45	0.001	-1.20	0.001
pyrF	1341921	1342658	orotidine-5'-phosphate decarboxylase	1.21	0.000	1.53	0.000	0.84	0.068	0.88	0.019
yciH	1342658	1342984	putative translation factor	1.28	0.000	1.57	0.000	0.87	0.084	0.72	0.126
trkG	1423782	1425239	Rac prophage; K+ transporter TrkG	0.21	0.396	0.42	0.033	0.23	0.501	0.20	0.574
fdnG	1547401	1550448	formate dehydrogenase N-subunit	-1.62	0.000	-1.94	0.000	-1.20	0.022	-1.21	0.001
pqqL	1572407	1575202	periplasmic metalloprotease	2.00	0.000	-0.61	0.002	-0.98	0.000	-0.52	0.126
yddB	1575247	1577619	putative TonB-dependent receptor YddB	2.93	0.000	-0.43	0.032	-0.92	0.000	-0.41	0.245
yddA	1577657	1579342	ABC transporter family protein YddA	3.65	0.000	0.59	0.037	-0.18	0.766	0.01	0.989
ydeT	1588853	1590001	fimbrial usher domain-containing protein YdeT	1.54	0.000	1.40	0.000	0.98	0.072	0.84	0.089
ynfO	1636756	1636989	Qin prophage; DUF3950 domain-containing protein YnfO	-0.97	NA	-1.52	0.005	-0.62	NA	-0.89	NA
ydfO	1637047	1637457	Qin prophage; DUF1398 domain-containing protein YdfO	-1.22	0.013	-1.51	0.001	-1.31	0.008	-0.90	NA
ynfE	1658069	1660495	putative selenate reductase YnfE	-1.47	0.000	-1.96	0.000	-1.13	0.034	-1.08	0.034
ynfK	1666524	1667219	putative dethiobiotin synthetase	-1.85	0.000	-1.82	0.000	-1.26	0.007	-1.55	0.000
Nth	1711523	1712158	endonuclease III	-0.23	0.603	-0.11	0.780	-0.12	0.799	-0.51	0.200
dtpA	1712769	1714271	dipeptide/tripeptide:H+ symporter DtpA	-0.17	0.460	0.26	0.340	-0.02	0.955	-0.14	0.843
purR	1737844	1738869	DNA-binding transcriptional repressor PurR	1.00	0.001	1.54	0.000	0.41	0.535	0.88	0.063
ydhY	1753851	1754477	putative 4Fe-4S ferredoxin-like protein YdhY	-1.84	0.000	-2.03	0.000	-1.22	0.021	-1.34	0.004
yoaC	1894133	1894432	DUF1889 domain-containing protein YoaC	0.06	0.899	0.30	0.421	0.79	0.027	0.62	0.088
yobF	1907448	1907591	DUF2527 domain-containing protein YobF	-0.55	0.051	-0.31	0.060	0.08	0.803	-0.53	0.087
yebY	1923365	1923706	DUF2511 domain-containing protein YebY	-0.19	0.383	0.02	0.962	-0.63	0.007	-0.07	0.883
yebZ	1923719	1924591	putative inner membrane protein	-0.20	0.582	-0.04	0.910	-0.84	0.004	-0.14	0.799
yobA	1924595	1924969	CopC domain-containing protein	-0.39	0.159	-0.30	0.286	-1.04	0.000	-0.25	0.556
purT	1930881	1932059	phosphoribosylglycinamide formyltransferase 2	1.02	0.093	1.89	0.001	0.04	0.958	1.28	0.024
flhC	1977266	1977844	DNA-binding transcriptional dual regulator FlhC	0.33	0.098	0.16	0.486	0.08	0.828	-0.08	0.892
flhD	1977847	1978197	DNA-binding transcriptional dual regulator FlhD	0.40	0.067	0.17	0.503	0.10	0.792	-0.03	0.970
isrC	2071317	2071511	CP4-44 prophage; small RNA IsrC	4.47	0.000	4.21	0.000	3.76	0.000	0.22	0.832
Flu	2071539	2074658	CP4-44 prophage; self recognizing antigen 43 (Ag43) autotransporter	4.56	0.000	4.33	0.000	3.83	0.000	0.10	0.929
yeeR	2074779	2076311	CP4-44 prophage; inner membrane protein YeeR	4.31	0.000	4.07	0.000	3.89	0.000	0.11	0.924
yeeS	2076308	2076754	CP4-44 prophage; RadC-like JAB domain-containing protein YeeS	3.60	0.000	3.51	0.000	3.31	0.000	0.08	0.947
yeeT	2076817	2077038	CP4-44 prophage; DUF987 domain-containing protein YeeT	1.62	NA	1.37	0.027	1.23	NA	-0.10	NA

Table S5: RNA-seq data of LTTRs

Gene	pos 1	pos 2	Function	WT/ΔlrhA log2 (FC)	P-adj	WT/ΔyafC log2 (FC)	P-adj	WT/ΔydcI log2 (FC)	P-adj	WT/ΔyhaJ log2 (FC)	P-adj
yehC	2190926	2191645	putative fimbrial chaperone YehC	-1.28	NA	-1.82	0.001	-0.91	NA	-0.98	NA
yehD	2191680	2192222	putative fimbrial protein YehD	-1.68	0.000	-2.33	0.000	-1.20	0.030	-1.09	0.037
napG	2299565	2300260	ferredoxin-type protein NapG	-1.26	0.001	-1.69	0.000	-1.10	0.012	-0.89	0.047
napA	2300267	2302753	periplasmic nitrate reductase subunit NapA	-1.43	0.000	-2.01	0.000	-1.26	0.006	-1.09	0.013
napD	2302750	2303013	NapA signal peptide-binding chaperone NapD	-1.42	0.000	-1.56	0.000	-1.16	0.022	-1.27	0.001
napF	2303003	2303497	ferredoxin-type protein	-1.77	0.000	-1.87	0.000	-1.32	0.005	-1.66	0.000
mqo	2305108	2306754	malate:quinone oxidoreductase	0.34	0.236	0.55	0.038	-1.24	0.000	0.08	0.913
purF	2428721	2430238	Amidophosphoribosyltransferase	1.30	0.003	2.13	0.000	0.22	0.784	1.40	0.003
cvpA	2430275	2430763	colicin V production protein	1.34	0.001	2.06	0.000	0.33	0.665	1.41	0.002
ypdK	2496921	2496992	uncharacterized membrane protein YpdK	1.33	0.000	1.70	0.000	1.06	0.012	0.97	0.035
valU	2520931	2521006	tRNA-Val	1.57	0.000	2.12	0.000	1.10	0.015	1.31	0.000
valX	2521051	2521126	tRNA-Val	1.62	0.000	1.90	0.000	1.11	0.007	1.30	0.000
valY	2521173	2521248	tRNA-Val	1.45	0.000	2.17	0.000	1.06	0.029	1.18	0.012
ypfM	2590807	2590866	uncharacterized protein YpfM	-1.31	0.000	-1.84	0.000	-1.31	0.002	-1.35	0.001
purC	2596905	2597618	phosphoribosylaminoimidazole-succinocarboxamide synthase	1.14	0.046	1.76	0.001	0.16	0.828	1.55	0.001
purM	2621197	2622234	phosphoribosylformylglycinamide cyclo-ligase	1.19	0.036	2.25	0.000	0.20	NA	1.15	0.049
purN	2622234	2622872	phosphoribosylglycinamide formyltransferase 1	1.35	0.001	1.94	0.000	0.19	0.804	1.37	0.002
guaB	2632604	2634070	inosine 5'-monophosphate dehydrogenase	1.41	0.010	1.75	0.003	0.13	0.840	1.20	0.027
ndk	2644433	2644864	nucleoside diphosphate kinase	1.85	0.000	1.69	0.000	0.56	0.361	0.64	0.306
csiE	2665435	2666715	stationary phase-inducible protein CsiE	-1.85	0.000	-0.11	0.665	0.11	0.789	0.18	0.687
purL	2691656	2695543	phosphoribosylformylglycinamide synthetase	1.22	0.008	1.72	0.001	0.21	0.787	1.34	0.006
grcA	2716066	2716449	stress-induced alternate pyruvate formate-lyase subunit	-1.48	0.000	-1.71	0.000	-1.02	0.040	-1.04	0.025
raiA	2737154	2737495	ribosome-associated inhibitor A	-1.84	0.000	-1.98	0.000	-1.60	0.000	-1.42	0.000
ypjF	2777453	2777782	CP4-57 prophage; toxin of the YpjF-YfjZ toxin-antitoxin system	0.57	0.280	1.34	0.002	1.10	0.024	1.51	0.001
glaH	2788985	2789962	glutarate dioxygenase GlaH	-0.74	0.249	-1.63	0.000	-0.70	0.212	-0.62	0.200
lhgD	2789982	2791250	L-2-hydroxyglutarate dehydrogenase	-1.12	0.012	-1.80	0.000	-0.80	0.136	-0.82	0.037
gabD	2791273	2792721	succinate-semialdehyde dehydrogenase (NADP+) GabD	-1.26	0.001	-2.02	0.000	-0.88	0.105	-0.98	0.012
gabT	2792735	2794015	4-aminobutyrate aminotransferase GabT	-1.20	0.001	-2.00	0.000	-0.82	0.144	-0.92	0.024
gabP	2794253	2795653	4-aminobutanoate:H+ symporter	-1.18	0.003	-2.05	0.000	-0.80	0.159	-0.88	0.054
stpA	2798091	2798495	DNA-binding transcriptional repressor StpA with RNA chaperone activity	2.18	0.000	2.45	0.000	0.92	0.106	1.62	0.000
argV	2818473	2818549	tRNA-Arg (ACG)	1.35	0.000	1.57	0.000	1.01	0.010	1.03	0.000
serV	2818553	2818645	tRNA-Ser (GCU)	1.20	0.000	1.52	0.000	1.14	0.001	1.15	0.000
gutM	2828621	2828980	DNA-binding transcriptional activator GutM	-0.10	0.848	-0.04	0.945	-0.23	0.669	-0.23	0.702
hypA	2850647	2850997	hydrogenase 3 nickel incorporation protein HypA	-1.28	0.000	-1.51	0.000	-1.29	0.003	-1.32	0.001
hypB	2851001	2851873	hydrogenase isoenzymes nickel incorporation protein HypB	-1.37	0.000	-1.82	0.000	-1.35	0.001	-1.44	0.000
cysJ	2890099	2891898	sulfite reductase, flavoprotein subunit	1.03	0.016	1.31	0.000	0.76	0.174	1.25	0.015
queD	2892214	2892579	6-carboxy-5,6,7,8-tetrahydropterin synthase	0.73	0.037	0.90	0.000	0.72	0.064	0.38	0.503
gudD	2918045	2919385	D-glucarate dehydratase	-1.11	0.013	-1.90	0.000	-1.05	0.039	-0.87	0.125
gudX	2919406	2920746	glucarate dehydratase-related protein	-1.48	0.007	-3.03	0.000	-1.08	0.064	-0.93	0.113
gudP	2920748	2922100	galactarate/D-glucarate transporter GudP	-4.44	0.000	-4.80	0.000	-1.23	0.024	-3.39	0.000

Table S5: RNA-seq data of LTTRs

Gene	pos 1	pos 2	Function	WT/ΔlrhA log2 (FC)	P-adj	WT/ΔyafC log2 (FC)	P-adj	WT/ΔydcI log2 (FC)	P-adj	WT/ΔyhaJ log2 (FC)	P-adj
ygeW	3006262	3007452	putative carbamoyltransferase YgeW	-3.26	0.000	-3.42	0.000	-1.13	0.046	-1.30	0.021
ygeX	3007510	3008706	2,3-diaminopropionate ammonia-lyase	-2.30	0.000	-2.62	0.000	-1.48	0.005	-1.48	0.004
ygeY	3008764	3009975	putative peptidase YgeY	-1.39	0.000	-1.65	0.000	-1.07	0.015	-0.91	0.053
yqeB	3012614	3014239	XdhC-CoxI family protein YqeB	-1.21	0.002	-1.57	0.000	-0.82	0.156	-0.91	0.082
yqeC	3014287	3015057	uncharacterized protein YqeC	-1.37	0.001	-1.79	0.000	-0.96	0.095	-1.19	0.014
mocA	3015160	3015738	molybdenum cofactor cytidylyltransferase	-1.60	0.000	-1.57	0.000	-0.79	0.136	-1.23	0.001
ygfK	3016060	3019158	putative oxidoreductase, Fe-S subunit	-2.96	0.000	-3.29	0.000	-1.19	0.038	-1.16	0.045
ssnA	3019161	3020489	putative aminohydrolase SsnA	-1.59	0.000	-1.98	0.000	-0.96	0.088	-1.07	0.035
uacT	3031367	3032815	urate:H+ symporter	-1.97	0.000	-1.97	0.000	-1.07	0.023	-1.11	0.019
yqfA	3042489	3043148	hemolysin-III family protein YqfA	-1.28	0.000	-1.60	0.000	-1.16	0.010	-1.33	0.002
ansB	3099682	3100728	L-asparaginase 2	-2.54	0.000	-3.33	0.000	-1.30	0.022	-2.17	0.000
glcG	3123827	3124231	putative heme-binding protein GlcG	-0.67	0.024	-1.58	0.000	-0.81	0.084	-0.72	0.064
glcF	3124236	3125459	glycolate dehydrogenase, putative iron-sulfur subunit	-0.95	0.011	-1.78	0.000	-0.81	0.136	-1.11	0.002
glcE	3125470	3126522	glycolate dehydrogenase, putative FAD-binding subunit	-1.48	0.000	-2.43	0.000	-1.23	0.022	-1.62	0.000
glcD	3126522	3128021	glycolate dehydrogenase, putative FAD-linked subunit	-1.55	0.000	-2.43	0.000	-1.22	0.024	-1.67	0.000
ygiZ	3171879	3172211	DUF2645 domain-containing inner membrane protein YgiZ	0.37	NA	0.53	0.484	0.28	NA	0.11	NA
mdaB	3172530	3173111	NADPH:quinone oxidoreductase MdaB	0.49	0.065	0.89	0.000	0.14	0.798	0.52	0.088
ttdR	3205324	3206256	Dan transcriptional activator // DNA-binding transcriptional activator Dan	-2.14	0.000	-2.04	0.000	-1.35	0.006	-1.63	0.000
rpsU	3210781	3210996	30S ribosomal subunit protein S21	1.50	0.000	1.69	0.000	0.94	0.047	1.04	0.004
yqjF	3250562	3250954	DoxX family protein	-0.01	0.985	0.04	0.955	0.21	0.746	-1.83	0.000
tdcB	3265039	3266028	catabolic threonine dehydratase	-1.13	0.053	-1.84	0.000	-1.54	0.000	-1.29	0.008
tdcA	3266127	3267065	DNA-binding transcriptional activator TdcA	-2.05	0.000	-2.11	0.000	-1.50	0.003	-1.87	0.000
garK	3270625	3271770	glycerate 2-kinase 1	-1.17	0.012	-1.85	0.000	-0.99	0.068	-0.69	0.177
garR	3271867	3272751	tartronate semialdehyde reductase	-1.30	0.004	-2.13	0.000	-1.21	0.025	-0.91	0.096
garL	3272787	3273557	alpha-dehydro-beta-deoxy-D-glucarate aldolase	-1.65	0.003	-3.69	0.000	-1.20	0.032	-1.24	0.031
garP	3273573	3274907	galactarate/D-glucarate transporter GarP	-4.12	0.000	-5.22	0.000	-1.16	0.029	-2.21	0.000
garD	3275282	3276853	galactarate dehydratase GarD	-1.70	0.000	-2.01	0.000	-1.21	0.014	-1.32	0.001
ubiU	3301485	3302480	ubiquinone biosynthesis protein UbiU	-1.98	0.000	-2.07	0.000	-1.53	0.004	-1.76	0.000
ubiV	3302489	3303367	ubiquinone biosynthesis protein UbiV	-1.74	0.000	-2.25	0.000	-1.30	0.012	-1.26	0.006
yhcC	3353121	3354050	radical SAM family oxidoreductase YhcC	-2.00	0.000	-2.04	0.000	-1.28	0.014	-1.64	0.000
yhdV	3418390	3418611	lipoprotein YhdV	-0.63	0.044	-0.13	0.757	0.01	0.981	-0.25	0.634
yhdX	3420134	3421315	putative ABC transporter membrane subunit YhdX	-0.33	0.336	0.25	0.504	0.90	0.024	0.10	0.898
yhdY	3421325	3422428	putative ABC transporter membrane subunit YhdY	-0.30	0.487	0.16	0.721	0.88	0.032	0.55	0.217
yhdZ	3422436	3423194	putative ABC transporter membrane subunit YhdZ	-0.19	0.615	-0.23	0.482	0.13	0.784	0.22	0.672
nirB	3494011	3496554	nitrite reductase catalytic subunit NirB	-2.57	0.000	-3.12	0.000	-1.10	0.053	-1.35	0.016
nirD	3496551	3496877	nitrite reductase subunit NirD	-1.66	0.001	-2.37	0.000	-1.31	0.019	-1.17	0.036
nikA	3613667	3615241	Ni (2+) ABC transporter periplasmic binding protein	-1.56	0.004	-2.14	0.000	-1.10	0.058	-1.85	0.000
nikB	3615241	3616185	Ni (2+) ABC transporter membrane subunit NikB	-2.14	0.000	-2.78	0.000	-1.57	0.003	-1.73	0.000
nikC	3616182	3617015	Ni (2+) ABC transporter membrane subunit NikC	-2.16	0.000	-2.82	0.000	-1.44	0.006	-1.75	0.000
nikD	3617015	3617779	Ni (2+) ABC transporter ATP binding subunit NikD	-2.09	0.000	-2.61	0.000	-1.51	0.003	-1.54	0.001

Table S5: RNA-seq data of LTTRs

Gene	pos 1	pos 2	Function	WT/ΔlrhA log2 (FC)	P-adj	WT/ΔyafC log2 (FC)	P-adj	WT/ΔydcI log2 (FC)	P-adj	WT/ΔyhaJ log2 (FC)	P-adj
nikE	3617776	3618582	Ni (2+) ABC transporter ATP binding subunit NikE	-1.80	0.000	-2.31	0.000	-1.31	0.014	-1.34	0.006
gadE	3658366	3658893	DNA-binding transcriptional activator GadE	-1.58	0.000	-0.08	0.879	0.02	0.979	-0.09	0.917
xylA	3729443	3730765	xylose isomerase	1.65	0.000	1.54	0.000	0.56	0.380	1.56	0.000
ysaA	3741109	3741582	putative electron transport protein YsaA	-1.72	0.000	-1.90	0.000	-1.25	0.016	-1.53	0.000
lldP	3777399	3779054	lactate/glycolate:H+ symporter LldP	-0.67	0.144	-0.56	0.071	-0.84	0.010	-0.40	0.296
lldR	3779054	3779830	DNA-binding transcriptional dual regulator LldR	-0.87	0.019	-0.69	0.009	-0.90	0.024	-0.62	0.014
lldD	3779827	3781017	L-lactate dehydrogenase	-1.03	0.001	-1.06	0.000	-1.07	0.010	-0.72	0.043
pyrE	3815127	3815768	orotate phosphoribosyltransferase	1.66	0.000	2.04	0.000	0.68	0.248	1.17	0.007
yicG	3818874	3819491	PF03458 family inner membrane protein YicG	0.64	0.024	0.66	0.000	-0.06	0.908	0.65	0.016
xanP	3828945	3830336	xanthine:H+ symporter XanP	0.90	0.134	2.12	0.000	0.15	NA	1.13	0.044
ilvN	3850802	3851092	acetohydroxy acid synthase I subunit IlvN	-1.24	0.001	-1.62	0.000	-0.94	0.099	-1.13	0.004
asnA	3927155	3928147	asparagine synthetase A	-1.30	0.000	-1.65	0.000	-1.40	0.000	-1.09	0.003
leuT	3982606	3982692	tRNA-Leu (CAG)	1.23	0.003	1.72	0.000	1.24	0.002	0.73	0.182
alaT	4037260	4037335	L-alanyl-tRNAalaT // tRNA-Ala (UGC)	1.14	NA	1.54	0.011	1.03	NA	0.22	NA
ptsA	4139720	4142221	putative PTS multiphosphoryl transfer protein PtsA	-1.15	0.000	-1.61	0.000	-1.38	0.000	-1.17	0.003
tyrU	4175472	4175556	tRNA-Tyr (GUA)	1.54	0.000	1.72	0.000	0.87	0.059	1.15	0.000
glyT	4175673	4175747	tRNA-Gly (UCC)	1.51	0.000	1.70	0.000	0.95	0.025	1.07	0.001
thrT	4175754	4175829	tRNA-Thr (GGU)	1.62	0.000	1.76	0.000	0.81	0.102	1.10	0.004
purH	4205943	4207532	bifunctional AICAR transformylase/IMP cyclohydrolase	0.89	0.147	1.64	0.003	0.08	NA	0.92	0.144
ghxP	4278479	4279828	guanine/hypoxanthine transporter GhxP	1.61	0.000	2.48	0.000	0.52	0.439	1.36	0.007
nrfA	4287764	4289200	cytochrome c552 nitrite reductase	-1.96	0.000	-2.45	0.000	-1.53	0.002	-1.53	0.000
nrfB	4289245	4289811	periplasmic nitrite reductase penta-heme c-type cytochrome	-1.61	0.000	-2.27	0.000	-1.39	0.007	-1.32	0.010
nrfC	4289808	4290479	putative menaquinol-cytochrome c reductase 4Fe-4S subunit	-1.33	0.007	-2.18	0.000	-1.25	0.020	-0.96	0.087
nrfD	4290476	4291432	putative menaquinol-cytochrome c reductase subunit NrfD	-1.17	0.003	-1.57	0.000	-0.84	0.124	-0.79	0.109
gltP	4294481	4295794	glutamate/aspartate: H+ symporter GltP	-0.78	0.000	-0.97	0.000	-1.02	0.002	-0.72	0.024
nrdD	4460522	4462660	anaerobic ribonucleoside-triphosphate reductase	-1.13	0.009	-1.57	0.000	-1.00	0.051	-0.73	0.206
pyrI	4470986	4471447	aspartate carbamoyltransferase, PyrI subunit	1.33	0.018	1.65	0.005	0.61	0.349	1.55	0.001
pyrB	4471460	4472395	aspartate carbamoyltransferase catalytic subunit	1.39	0.014	1.68	0.004	0.59	0.365	1.15	0.050
fimA	4543115	4543663	type 1 fimbriae major subunit	-2.04	0.000	-0.51	0.191	-0.52	0.317	-0.31	0.612
fimI	4543728	4544267	putative fimbrial protein FimI	-1.89	0.000	-0.16	0.599	-0.12	0.804	-0.10	0.883
fimC	4544304	4545029	type 1 fimbriae periplasmic chaperone	-1.76	0.000	-0.18	0.600	-0.14	0.750	-0.11	0.883
fimD	4545096	4547732	type I fimbriae usher protein	-1.79	0.000	-0.02	0.968	-0.06	0.918	-0.03	0.974
fimF	4547742	4548272	type 1 fimbriae minor subunit FimF	-1.92	0.000	-0.28	0.480	-0.37	0.472	-0.28	0.595
fimG	4548285	4548788	type 1 fimbriae minor subunit FimG	-1.72	0.000	-0.13	0.791	-0.14	0.811	0.07	0.933
yjiS	4571751	4571915	DUF1127 domain-containing protein YjiS	0.13	NA	0.28	NA	0.00	NA	0.23	NA
yjiT	4572414	4573931	putative uncharacterized protein YjiT	-0.51	0.001	-0.37	0.013	-0.32	0.149	-0.17	0.566
leuV	4606079	4606165	tRNA-Leu (CAG)	1.15	0.008	1.59	0.000	1.09	0.010	0.82	0.078
leuP	4606200	4606286	tRNA-Leu (CAG)	1.58	0.000	1.58	0.000	1.23	0.001	0.82	0.100

Table S5: RNA-seq data of LTTRs

Gene	pos 1	pos 2	Function	WT/ΔlrhA log2 (FC)	P-adj	WT/ΔyafC log2 (FC)	P-adj	WT/ΔydcI log2 (FC)	P-adj	WT/ΔyhaJ log2 (FC)	P-adj
leuQ	4606315	4606401	tRNA-Leu (CAG)	1.19	0.001	1.59	0.000	1.10	0.010	1.04	0.018
yjjW	4614680	4615543	putative glycyl-radical enzyme activating enzyme YjjW	-1.01	0.015	-1.51	0.000	-0.91	0.060	-0.88	0.074
yjjI	4615515	4617065	DUF3029 domain-containing protein YjjI	-2.11	0.000	-2.63	0.000	-1.74	0.000	-1.71	0.000

The log2 fold change (FC) values marked in green are specific for the respective LTTR as compared to other LTTRs. The cut off for log2 fold change (FC) for all LTTRs is taken as > (+/-)1.5.

The log2 fold change (FC) and P-adj values marked in grey have P-adj > 0.05 or NA (Not available).

The log2 fold change (FC) values in blue highlight the targets which are identified from ChIP-exo analysis (Gao *et al.*, 2018). The presumptive YdcI target genes corresponding to the log2 fold change (FC) values enclosed in black bold frames have strong peaks in IGV analysis whereas the log2 fold change (FC) values in black frames correspond to targets having weak peaks in IGV analysis.

References

Baba, T., Ara, T., Hasegawa, M., Takai, Y., Okumura, Y., Baba, M., Datsenko, K.A., Tomita, M., Wanner, B.L., and Mori, H. (2006) Construction of Escherichia coli K-12 in-frame, single-gene knockout mutants: the Keio collection. *Mol. Syst. Biol.* **2**: 2006.0008.

Bougdour, A., and Gottesman, S. (2007) ppGpp regulation of RpoS degradation via anti-adaptor protein IraP. *Proc. Natl. Acad. Sci. USA*: 0705561104.

Breddermann, H., and Schnetz, K. (2017) Activation of *leuO* by LrhA in *Escherichia coli*. *Mol. Microbiol.* **104**: 664-676.

Cherepanov, P.P., and Wackernagel, W. (1995) Gene disruption in Escherichia coli: TcR and KmR cassettes with the option of Flp-catalyzed excision of the antibiotic-resistance determinant. *Gene* **158**: 9-14.

Datsenko, K.A., and Wanner, B.L. (2000) One-step inactivation of chromosomal genes in *Escherichia coli* K-12 using PCR products. *Proc. Natl. Acad. Sci. USA* **97**: 6640-6645.

Fragel, S.M., Montada, A., Heermann, R., Baumann, U., Schacherl, M., and Schnetz, K. (2019) Characterization of the pleiotropic LysR-type transcription regulator LeuO of *Escherichia coli*. *Nucleic Acids Res.* **47**: 7363-7379.

Gao, Y., Yurkovich, J.T., Seo, S.W., Kabimoldayev, I., Dräger, A., Chen, K., Sastry, A.V., Fang, X., Mih, N., Yang, L., Eichner, J., Cho, B.-K., Kim, D., and Palsson, B.O. (2018) Systematic discovery of uncharacterized transcription factors in *Escherichia coli* K-12 MG1655. *Nucleic Acids Res.* **46**: 10682-10696.

Miroux, B., and Walker, J.E. (1996) Over-production of proteins in *Escherichia coli*: mutant hosts that allow synthesis of some membrane proteins and globular proteins at high levels. *J. Mol. Biol.* **260**: 289-298.

Pannen, D., Fabisch, M., Gausling, L., and Schnetz, K. (2016) Interaction of the RcsB response regulator with auxiliary transcription regulators in *Escherichia coli*. *J. Biol. Chem.* **291**: 2357-2370.

Rangarajan, A.A., and Schnetz, K. (2018) Interference of transcription across H-NS binding sites and repression by H-NS. *Mol. Microbiol.* **108**: 226-239.

Trautinger, B.W., Jaktaji, R.P., Rusakova, E., and Lloyd, R.G. (2005) RNA Polymerase Modulators and DNA Repair Activities Resolve Conflicts between DNA Replication and Transcription. *Mol. Cell* **19**: 247-258.

Uzzau, S., Figueroa-Bossi, N., Rubino, S., and Bossi, L. (2001) Epitope tagging of chromosomal genes in *Salmonella*. *Proc. Natl. Acad. Sci. USA* **98**: 15264-15269.

Yilmaz, C., Rangarajan, A.A., and Schnetz, K. (2020) The transcription regulator and c-di-GMP phosphodiesterase PdeL represses motility in *Escherichia coli*. *J. Bacteriol.* **203**: JB.00427-00420.

Zhang, A., Rimsky, S., Reaban, M.E., Buc, H., and Belfort, M. (1996) *Escherichia coli* protein analogs StpA and H-NS: regulatory loops, similar and disparate effects on nucleic acids dynamics. *EMBO J.* **15**: 1340-1349.
